# The Ability of a Charophyte Alga Hexokinase to Restore Glucose Signaling and Glucose Repression of Gene Expression in a Glucose-Insensitive *Arabidopsis* Hexokinase Mutant Depends on Its Catalytic Activity

**DOI:** 10.3389/fpls.2018.01887

**Published:** 2018-12-20

**Authors:** Mikael Ulfstedt, Guo-Zhen Hu, D. Magnus Eklund, Hans Ronne

**Affiliations:** ^1^Department of Forest Mycology and Plant Pathology, Swedish University of Agricultural Sciences, Uppsala, Sweden; ^2^Department of Plant Ecology and Evolution, Evolutionary Biology Centre, Uppsala University, Uppsala, Sweden

**Keywords:** hexokinase, glucose repression, *Arabidopsis thaliana*, *Klebsormidium nitens*, *Saccharomyces cerevisiae*, glucose signaling

## Abstract

Hexokinases is a family of proteins that is found in all eukaryotes. Hexokinases play key roles in the primary carbon metabolism, where they catalyze the phosphorylation of glucose and fructose, but they have also been shown to be involved in glucose signaling in both yeast and plants. We have characterized the *Klebsormidium nitens KnHXK1* gene, the only hexokinase-encoding gene in this charophyte alga. The encoded protein, KnHXK1, is a type B plant hexokinase with an N-terminal membrane anchor localizing the protein to the mitochondrial membranes. We found that KnHXK1 expressed in *Arabidopsis thaliana* can restore the glucose sensing and glucose repression defects of the glucose-insensitive hexokinase mutant *gin2-1*. Interestingly, both functions require a catalytically active enzyme, since an inactive double mutant was unable to complement *gin2-1*. These findings differ from previous results on *Arabidopsis* AtHXK1 and its orthologs in rice, where catalytic and glucose sensing functions could be separated, but are consistent with recent results on the rice cytoplasmic hexokinase OsHXK7. A model with both catalytic and non-catalytic roles for hexokinases in glucose sensing and glucose repression is discussed.

## Introduction

For all cells, whether they exist as single cells or as a part of a multicellular organism, it is vital to be able to sense if there are nutrients available that can sustain their metabolism and growth. Sugars are important as carbon and energy sources, but they also have regulatory roles in many organisms, controlling metabolism, stress resistance, growth and development (Rolland et al., 2001). In the yeast *Saccharomyces cerevisiae*, the favored carbon and energy source glucose is sensed by several pathways. One is the glucose repression pathway, which involves the Snf1 protein kinase, the major hexokinase Hxk2 and the glucose repressor Mig1 (Entian, 1980; Ronne, 1995; Hohmann et al., 1999; Johnston, 1999; Rolland et al., 2002). Snf1 is activated in the absence of glucose and phosphorylates both Hxk2 and Mig1, which causes their exclusion from the nucleus. In the presence of glucose, Snf1 is less active, and Hxk2 and Mig1 can move into the nucleus and form a repressing complex with Snf1 on the promoters of glucose-repressed genes (Ahuatzi et al., 2004, 2007; Fernández-García et al., 2012). Within this complex, Hxk2 masks a serine in Mig1 that is phosphorylated by Snf1, thus protecting Mig1 from phosphorylation and nuclear export (Pelaez et al., 2010).

This regulatory function of Hxk2 is independent of its catalytic activity as shown by mutations that abolish either activity. Thus, the Hxk2_wca_ mutant (without catalytic activity), with a substitution of serine-304 to a phenylalanine and a small C-terminal truncation, is catalytically inactive but mediates glucose repression. In contrast, the Hxk2_wrf_ mutant (without regulatory function), which lacks amino acids 6–15 in the N-terminus, is catalytically active but unable to mediate glucose repression (Pelaez et al., 2010). The Hxk2_wrf_ mutant is also unable to bind to Mig1, indicating that this interaction is important for the regulatory role of Hxk2. How Snf1 is inactivated in the presence of glucose is not fully understood, but Snf1 binds ADP which is generated under low energy conditions such as the absence of glucose (Mayer et al., 2011). This interaction protects Snf1 against dephosphorylation and inactivation by protein phosphatase 1. Accordingly, Snf1 is regulated by the cell’s energy level, similar to its animal homolog AMPK, the AMP-activated protein kinase (Carling, 2004).

Also plant hexokinases have such a dual role (Rolland et al., 2006; Claeyssen and Rivoal, 2007; Li and Sheen, 2016; Aguilera-Alvarado and Sanchez-Nieto, 2017). Early experiments in *Arabidopsis thaliana* suggested that hexokinase is involved in both glucose signaling and glucose metabolism (Jang and Sheen, 1994; Jang et al., 1997; Sheen et al., 1999). The notion that hexokinase is a sugar sensor in plants was, however, questioned by Halford et al. (1999a) who pointed out that alterations in hexokinase activity might simply affect the rate of metabolism of hexoses and therefore the production of ATP, and that the plant orthologs of Snf1/AMPK, the SnRK1 kinases, could mediate regulatory changes in response to altered ATP levels. See also further comments by Moore and Sheen (1999) and Halford et al. (1999b).

Further support for a role of plant hexokinases in glucose sensing came from a screen for glucose insensitive mutants in *Arabidopsis* (Leon and Sheen, 2003). One such mutant called *glucose insensitive 2-1* (*gin2-1*) is allelic to one of the hexokinase genes in *Arabidopsis, AtHXK1* (Moore et al., 2003). The *gin2-1* mutant has a nonsense mutation in *AtHXK1* that destabilizes the transcript and reduces the protein below detectable levels. It is defective in glucose sensing but not in glucose phosphorylation, which suggests that the other major hexokinase, AtHXK2, can provide catalytic activity but not the glucose sensing function of AtHXK1. Targeted mutagenesis of AtHXK1 showed that two mutations thought to cause catalytic inactivity, G104D and S177A, did not affect glucose sensing, thus providing support for the notion that the glucose sensing and catalytic activities of AtHXK1 can be separated (Moore et al., 2003). Consistent with this, the rice hexokinases OsHXK5 and OsHXK6 could complement the glucose signaling phenotype of the *gin2-1* mutant, and this ability was not affected when mutations corresponding to G104D and S177A were introduced into OsHXK5 and OsHXK6 (Cho et al., 2009).

It was subsequently reported that small amounts of AtHXK1 can translocate into the nucleus where it forms a complex with two other proteins that usually are found outside the nucleus: the VHA-B1 subunit of vacuolar H^+^-ATPase and the RPT5B subunit of the proteasome (Cho et al., 2006). Significantly, *vha-B1* and *rpt5b* have similar glucose insensitive phenotypes as *gin2*, including a defect in glucose repression of the *CAA* (carbonic anhydrase) and *CAB* (chlorophyll a/b binding protein) genes (Cho et al., 2006). This suggested that the nuclear complex might mediate glucose repression. Consistent with this, ChIP experiments showed that the AtHXK1 complex binds to the *CAB2* promoter. Furthermore, VHA-B1 was found to interact in a yeast 2-hybrid screen with two transcription factors, SCL3 and an unnamed MYB-like protein, and it was proposed that the complex may be recruited to glucose-responsive promoters by these and other transcription factors that bind to the complex (Cho et al., 2006). This model would be analogous to the role of the hexokinase nuclear complex in yeast.

There are, however, findings that are not easily explained by this model. Cyanobacterial glucokinase from *Synechocystis* can complement *gin2-1* with respect to both glucose-responsive growth phenotypes and glucose repression of the *CAB* and *RBCS* genes (Ryu et al., 2008). It is unlikely that cyanobacterial glucokinase could participate in specific protein-protein interactions in the nuclei of *Arabidopsis* cells since this prokaryotic enzyme is very distantly related to the eukaryotic hexokinases (Kawai et al., 2005). More recently, the rice cytoplasmic hexokinase OsHXK7 was shown to complement both the glucose sensing and glucose repression defects of *gin2-1*, but unlike OsHXK5 and OsHXK6, this ability was dependent on its catalytic activity (Kim et al., 2016). This suggests that two hexokinase-dependent glucose signaling pathways with similar or identical targets exist in plants, one that requires the catalytic activity of hexokinase, and one that does not.

An interesting question is if glucose signaling is found in all streptophytes, i.e., the land plants (embryophytes) and charophyte algae, or if it was a later development during the evolution of land plants. *Klebsormidium nitens* is a simple filamentous microalga that belong to the charophytes, the freshwater algae from which the land plants developed approximately 700 million years ago (Clarke et al., 2011; Graham et al., 2012). Studies of *K. nitens* may therefore provide insights into plant evolution. We here report the characterization of the single hexokinase present in *K. nitens*. The protein, KnHXK1, has an N-terminal membrane anchor and is related to the membrane-associated type B hexokinases found in the moss *Physcomitrella patens* and vascular plants (Olsson et al., 2003; Nilsson et al., 2011). Consistent with this, KnHXK1 localized to mitochondrial membranes when expressed in *Physcomitrella* protoplasts. We further found that KnHXK1 can complement the glucose insensitive phenotype of the *Arabidopsis gin2-1* mutant and restore glucose repression of the photosynthetic *CAA* and *CAB2* genes. Interestingly, a catalytically inactive KnHXK1 mutant was unable to complement the *gin2-1* mutation. These findings are consistent with recent results on the function of OsHXK7 (Kim et al., 2016) but differ from previous results with *Arabidopsis* AtHXK1 and its orthologs in rice (Moore et al., 2003; Cho et al., 2009). A model with both catalytic and non-catalytic roles for hexokinases in glucose sensing and glucose repression is discussed.

## Materials and Methods

### Plant Material and Growth Conditions

The *A. thaliana* wild type (Landsberg *erecta*, L*er*) and *gin2-1* mutant (in the L*er* background) were obtained from the *Arabidopsis* Biological Resource Centre (ABRC), Ohio State University, Columbus, OH, United States. The *P. patens* strain used was the Gransden Wood ecotype (Ashton and Cove, 1977). *K. nitens* strain SAG 52.91 was obtained from the SAG Culture Collection of Algae at University of Göttingen, Germany. The strain was originally isolated in a bog on Mors Island, Denmark. It should be noted that this is probably not the same species as the American *Klebsormidium* strain NIES-2285 or SAG 335-1a whose genome was sequenced by Hori et al. (2014). The latter was called *K. flaccidum* in the genome sequence paper, but was recently renamed to *K. nitens* (Ohtaka et al., 2017). We found that the genome sequence of the European *K. nitens* only could be partially aligned to that of the American strain, and that the sequences differ significantly within the aligned regions (unpublished). Based on this, we conclude that the European and American strains most likely are different species, even though both are annotated as *K. nitens* in the strain collections.

*Arabidopsis* was grown at 22°C and 110 μmol m^-2^ s^-1^ using a 16 h/8 h light/dark photoperiod. *K. nitens* and *P. patens* were grown on cellophane covered 0.8% agar plates containing BCD media (Nishiyama et al., 2000), supplemented with 5 mM ammonium tartrate. Plants were grown at 25°C, constant light, in a Sanyo MLR-350 light chamber using fluorescent tubes (FL40SS W/37, Toshiba) at about 30 μmol m^-2^ s^-1^. The ability of *K. nitens* to use different carbon sources was tested on ammonium tartrate supplemented BCD plates containing either 0.05 or 0.25 M glucose, fructose, sucrose, raffinose, galactose, or mannitol. The plates were incubated at 25°C with continuous light or in darkness for 1 month.

### Yeast Strains

All yeast strains were congenic to the *MAT*a *ade2-1 can1-100 his3-11,15 leu2-3,112 trp1-1 ura3-1* yeast strain W303-1A (Thomas and Rothstein, 1989). The *hxk2::LEU2* single knockout strain SH310 and the *hxk1::HIS3 hxk2::LEU2 glk1::LEU2* triple knockout strain SH 7.4.-3C (Hohmann et al., 1999) were kindly provided by Stefan Hohmann.

### Cloning of KnHXK1 cDNA and Vector Construction

The primers KnHxk1-F and KnHxk1-R (Table 1) were used to amplify a full length 1552 bp *KnHXK1* cDNA that was cloned into the TA-cloning vector pCR^®^ 2.1-TOPO, thus producing pKnHXK1 (Table 2). To generate a catalytically inactive mutant of KnHXK1 the conserved glycine (G) of the ATP binding site was mutated into an aspartic acid (D) using primer G110D (Table 1), and the conserved serine (S) in the phosphoryl transfer site was mutated into an alanine (A) using primer S184A (Table 1). Mutations were generated using a QuikChange^®^ Multi Site-Directed Mutagenesis Kit from Stratagene on plasmid pKnHXK1, generating plasmid pKnMu.

**Table 1:** Oligonucleotides used.

Name	Sequence
KnHXK1-F	5′-GATCGGCAAACATGTCTGATAATGG-3′
KnHXK1-R	5′-ACCTACTTTGCCTTGTACTTGGAG-3′
KnHXK1-GFP-F1	5′-GGATCCATGTCTGATAATGGAAGTCCGAG-3
KnHXK1-GFP-F2	5′-GGATCCATGGCTTACGCTGTGTATCAGAG-3′
KnHXK1-GFP-R2	5′-GGATCCGCTGCTTTGCCTTGTACTTGGA-3′
G110D	5′-GCGCTGGATCTGGGAGATACCAACTTTAGAGTC-3′
S184A	5′-CTCGGATTCACGTTCGCTTTCCCGTGCAACC-3′
CAB-F	5′-ATGGCCACTTCAGCAATCCAA-3′
CAB-R	5′-CACAACTTGACACGCCCATAT-3′
CAA-F	5′-TGAATACGCTGTCTTGCACC-3′
CAA-R	5′-TGTGATGGTGGTGGTAGCGA-3′
MUAraF2	5′- TTACGCTGTGTATCAGAGGGTCTACG-3′
MUAraR1	5′- CGGGTCAGAGCCTCAGTAAGCA-3′
UBQ-F	5′-GTGGTGCTAAGAAGAGGAAGA-3′
UBQ-R	5′-TCAAGCTTCAACTTCTTCTTT-3′
qCAB-F	5′-GCAATGGCCACTTCAGCAAT-3′
qCAB-R	5′-CTCCGATTTTGCGGAGGAGA-3′
qUBQ-F	5′-GATCGCTCTTCACATCTCTTCG-3′
qUBQ-R	5′-ACCAGTGAGTGTTTTCACGA-3′


**Table 2:** Plasmids used.

Plasmid	Insert
p35SSRDXG	See Mitsuda et al. (2006)
pBCKH	See Mitsuda et al. (2006)
pKnHXK1-GFP-F1	KnHXK1 (codons 1-512) fused to GFP
pKnHXK1-GFP-F2	KnHXK1 (codons 27-512) fused to GFP
pKnHXK1-GFP-F3	Catalytically inactive KnHXK1 (codons 1-512) fused to GFP
psmRS-GFP	GFP expression cassette (Davis and Vierstra, 1998)
pKnHXK1	KnHXK1 gene
pKnMu	KnHXK1 cDNA mutated G110D and S184A
pX-anch	KnHXK1 cDNA (codons 27-512)
p35SKnHxk1	KnHXK1 cDNA (codons 1-512) cloned into p35SSRDXG
p35SKnMu	KnHXK1 cDNA mutated G110D and S184A cloned into p35SSRDXG
p35SX-anch	KnHXK1 cDNA (codons 27-512) cloned into p35SSRDXG
pG35SKnHxk1	KnHXK1 cDNA (codons 1-512) under 35S promoter in pBCKH
pG35SKnMu	KnHXK1 cDNA (codons 1-512) mutated G110D and S184A under 35S promoter in pBCKH
pG35SX-anch	KnHXK1 cDNA (codons 27-512) under 35S promoter in pBCKH
pX-KnMu	KnHXK1 cDNA (codons 27-512) mutated G110D and S184A
pKnWT	KnHXK1 (codons 1-512) in pFL61
pKnNΔ	KnHXK1 (codons 27-512) in pFL61
pKnCI	KnHXK1 (codons 1-512) mutated G110D and S184A in pFL61
pKnNΔCI	KnHXK1 (codons 27-512) mutated G110D and S184A in pFL61
pFL61	*PGK* promoter, 2μ origin, *URA3* (Minet et al., 1992)


TargetP v1.1 (Emanuelsson et al., 2007) was used to predict the membrane anchor of KnHXK1. Primers KnHXK1-GFP-F2 and KnHXK1-GFP-R2 (Table 1) were used to amplify a 1477 bp *KnHXK1* fragment lacking the sequences encoding the membrane anchor (codons 1–26), with a methionine codon added instead in position 26. This PCR fragment was cloned into the TA-cloning vector pCR^®^ 2.1-TOPO, thus producing pX-anch (Table 2). pKnHXK1 and pKnMu were cut with *Bam*HI and *Xho*I, and pX-anch was cut with *Bam*HI, and the resulting fragments were blunted using the Klenow fragment (ThermoFisher Scientific). The vector p35SSRDXG (Mitsuda et al., 2006) was cut with *Sma*I and *Sal*I, and then blunted. The different fragments were then cloned into p35SSRDXG, producing plasmids p35SKnHXK1, p35SKnMu and p35SX-anch. Expression cassettes were subsequently moved to the binary plasmid pBCKH (Mitsuda et al., 2006) by recombination using the GATEWAY system, thus producing the plasmids pG35SKnHXK1, pG35SKnMu, and pG35SX-anch.

### Transformation of *Arabidopsis*

Binary plasmids were transformed into the *Agrobacterium* strain GV3101. The *gin2-1* plants were transformed using the floral dip method (Clough and Bent, 1998). Transformants were selected on MS medium containing 25 mg/l hygromycin. Homozygous single insertion lines were used for the subsequent phenotypic analysis.

### GFP Fusions and Localization in *Physcomitrella* Protoplasts

The plasmid psmRS-GFP (Davis and Vierstra, 1998) carries a 35S promoter in front of a soluble modified red shifted GFP followed by the *NOS1* terminator. Primers KnHXK1-GFP-F1 and KnHXK1-GFP-R2 (Table 1) were used to amplify a full length 1552 bp fragment that was cloned into the *Bam*HI site of psmRS-GFP, thus producing plasmid pKnHXK1-GFP-F1 (Table 2), encoding a KnHXK1-(1-512)-GFP fusion (KnWT-GFP). Primers KnHXK1-GFP-F2 and KnHXK1-GFP-R2 (Table 1) were used to amplify a 1477 bp fragment that was cloned into the *Bam*HI site of psmRS-GFP, thus producing plasmid pKnHXK1-GFP-F2 (Table 2), encoding a KnHXK1-(27-512)-GFP fusion without the KnHXK1 membrane anchor (KnNΔ-GFP). Primers KnHXK1-GFP-F1 and KnHXK1-GFP-R2 were also used to amplify a 1552 bp fragment from plasmid pKnMu, which was cloned as described above, producing plasmid pKnHXK1-GFP-F3 (Table 2), encoding a catalytically inactive KnHXK1-(1-512)-GFP fusion (KnCI-GFP).

Plasmids were transformed into *Physcomitrella* protoplasts by PEG-mediated transformation (Schaefer et al., 1991). Two days after transformation protoplasts were analyzed using a Zeiss Axioskop 2 fluorescence light microscope. The GFP signal was detected using a FITC filter (excitation 480 nm, emission 535 nm, and dichronic beamsplitter 505 nm) while chloroplast autofluorescence was detected using a TRITC filter (excitation 535 nm, emission 620 nm, and dichronic beamsplitter 565 nm).

### Yeast Complementation Experiments

For yeast complementation studies, a yeast strain containing knockouts of *HXK1, HXK2*, and *GLK1* in the W303-1A background was used (Hohmann et al., 1999). Hexokinase-encoding cDNA constructs from *K. nitens* excised from plasmids using either *Bam*HI and *Xho*I (pKnHXK1 and pKnMu) or *Bam*HI (pX-anch), were blunted and cloned into the high copy number 2 μm shuttle vector pFL61 (Minet et al., 1992), which expresses inserts in yeast from the constitutive *PGK* promoter, thus producing plasmids pKnWT, pKnNΔ, and pKnCI (Table 2). Primers KnHXK1-GFP-F2 and KnHXK1-GFP-R2 were used to amplify a fragment from pKnMu that was cloned into pCR^®^ 2.1-TOPO, thus producing pX-KnMu (Table 2). Hexokinase-encoding cDNA sequences from pX-KnMu were excised using *Bam*HI, blunted, and cloned into pFL61 thus producing pKnNΔCI (Table 2). Transformants were selected on synthetic uracil-less media containing 2% galactose in order to permit the *hxk1 hxk2 glk1* triple knockout strain, which cannot utilize glucose, to grow. Colonies were picked to synthetic galactose plates lacking uracil, and then diluted to OD 2.0 and 0.2 and spotted onto synthetic media lacking uracil but containing different carbon sources. Growth was scored after 4–6 days at 30°C. Some plates also contained 1 μg/ml ethidium bromide in order to inhibit non-fermentative growth on fermentable carbon sources. In the glucose repression assay, the transformants were spotted onto synthetic media lacking uracil, containing 2% glycerol, 2% ethanol, 0.05% glucose, and 8 mg/ml of 2-deoxyglucose (Kraakman et al., 1999).

### Secreted Invertase Assay in Yeast

Wild type yeast transformed with the vector pFL61, and a yeast *hxk2* knockout strain transformed with pFL61, pKnWT, and pKnNΔCI, respectively, were grown overnight in synthetic uracil-less media containing 2% glucose, diluted to OD 0.1 in fresh media and incubated for 2 h at 30°C. An aliquot of 1 × 10^6^ cells was then removed from each sample, washed 3× in water, and put on ice. Secreted invertase activity in each sample was assayed using an Invertase assay kit (MAK118, Sigma Aldrich), according to the manufacturer’s instructions. One unit of invertase is the amount of enzyme that will catalyze the formation of 1.0 μmol of glucose per minute at pH 4.5 under the assay conditions.

### Phenotypic Characterization of *Arabidopsis* Transformants

Transgenic *Arabidopsis* seedlings were grown on ½ MS medium containing 6% glucose or 6% mannitol for 6 days under 100 μmol m^-2^ s^-1^ light. For the high light experiment, transgenic *Arabidopsis* plants were grown on soil for 18 days under either low light conditions or high light conditions, 70 and 240 μmol m^-2^ s^-1^, respectively (Cho et al., 2009). Three different lines transformed with each construct were initially tested for glucose sensitivity (Table 3). The same lines are shown in Figures 6–9 in the same order as they appear in Table 3, expect for the KnCI construct where line 4 was replaced by line 1 in Figures 7–9 due to a lack of seeds.

**Table 3:** Growth inhibition on 6% glucose of *Arabidopsis* seedlings transformed with different *KnHXK1* constructs.

Line	Inhibition on 6% glucose	Inhibition on 6% mannitol
KnWT 8	100% (50/50)	0% (0/50)
KnWT 13	100% (50/50)	0% (0/50)
KnWT 19	100% (50/50)	0% (0/50)
KnCI 4	0% (0/15)	0% (0/13)
KnCI 8	0% (0/49)	0% (0/50)
KnCI 11	0% (0/38)	0% (0/17)
KnNΔ 5	100% (41/41)	0% (0/17)
KnNΔ 12	55% (11/20)	0% (0/12)
KnNΔ 13	63% (24/38)	0% (0/25)


*The number of seedlings that were scored and the fraction that was growth inhibited is shown for three lines transformed with each construct. Growth inhibition on 6% mannitol was also scored as a negative control.*

### Isolation of RNA and RT-PCR Analysis of Gene Expression

Total RNA from 6-day-old seedlings was isolated using the RNeasy^®^ Plant mini kit (Qiagen). The RNA was reverse transcribed using an iScript kit from Bio-Rad. cDNA from each sample was diluted to 6.5 ng/ul for use as template in the PCR. PCR on the resulting cDNA was performed using primers CAB-F and CAB-R (Table 1) for *CAB2* (At3g27690), and CAA-F and CAA-R (Table 1) for *CAA* (At5g14740). As an internal control, primers UBQ-F and UBQ-R (Table 1) were used to amplify cDNA from the *UBQ5* (At3g62250) gene (Moore et al., 2003). Expression of the *KnHXK1* mRNA in the transgenic lines was monitored using primers MUAraF2 and MUAraR1 (Table 1). The PCR protocol used was 2 min at 95°C, then 27 cycles of 95°C for 45 s, 53°C for 30 s, 72°C for 1 min and a single step of 5 min at 72°C. The kit used for PCR was the Taq DNA Polymerase, recombinant (5 U/μL) (EP0401) from ThermoFisher Scientific. The PCR products were separated on a 1,5% agarose gel.

### qPCR Analysis of Gene Expression

For quantitative RT-PCR (qPCR) we used the same cDNA as in the RT-PCR experiment. The PCR protocol used was 98°C for 1 min, then 40 cycles of 95°C for 5 s, 60°C for 5 s, followed by a meltcurve test in which the temperature was raised from 65 to 95°C in 0.5°C increments. The kit used for qPCR was the SsoFast^TM^ EvaGreen^®^ Supermix from Bio-Rad. Each reaction contained the following mix: 5 μl SsoFast^TM^ EvaGreen Supermix, 0.4 μl (10 μM) forward primer, 0.4 μl (10 μM) reverse primer, 2.2 μl H_2_O and 2 μl of cDNA template. The qPCR reactions were run on a Bio-Rad CFX Connect Real-Time System. The PCR primers used were qCAB-F and qCAB-R (Table 1) for CAB2 (At3g27690) and qUBQ-F and qUBQ-R (Table 1) for UBQ4 (At5g20620). Expression of the *UBQ4* gene was used for normalization.

### Sequence Analysis

The Vector NTI software package with ContigExpress (InforMax Inc., Bethesda, MD) was used for sequence editing, sequence analysis and building of contigs. To build the phylogenetic tree in Figure 2, we used the Neighbor-Joining method (Saitou and Nei, 1987) as previously described (Thelander et al., 2004).

## Results

### The Charophyte Alga *Klebsormidium nitens* Can Grow in the Dark on Externally Supplied Carbon Sources

The purpose of glucose repression in yeast is to adapt the cell for rapid growth on externally supplied glucose, typically obtained from grapes and other fruits, by turning off pathways used to metabolize alternative carbon sources. Hexokinase is important both for the initial metabolism of glucose and other sugars, and for glucose signaling and glucose repression. Plants, being autotrophs, are not dependent on any other carbon sources than the CO_2_ needed for carbon fixation. However, they can also utilize sugars and other organic compounds absorbed from the environment. Thus, we have previously shown that the moss *Physcomitrella* can grow on petri dishes containing glucose in the absence of light, and that this ability to use externally supplied glucose is dependent on the *PpHKX1* gene encoding the major hexokinase isozyme in *Physcomitrella* (Olsson et al., 2003).

**FIGURE 1:**
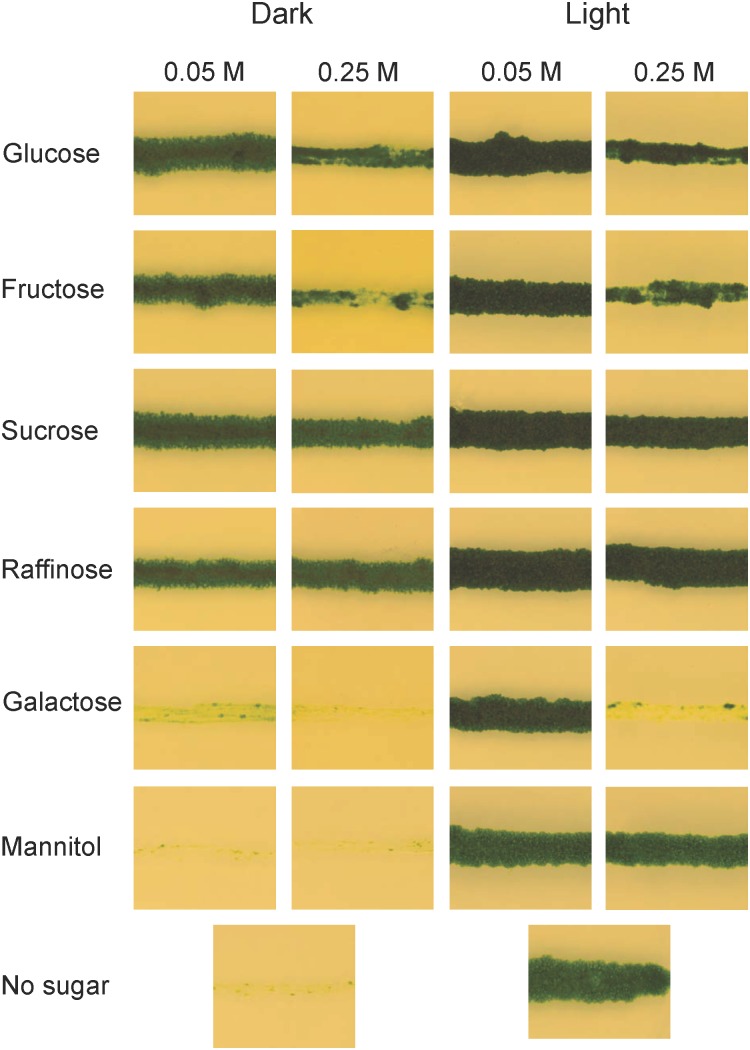
Growth of *Klebsormidium nitens* on different carbon sources in light as well as in darkness. *K. nitens* was grown on BCD media with two different concentrations of glucose, fructose, sucrose, raffinose, galactose, or mannitol for 1 month in light as well as in darkness. BCD without any carbon source was included as a control.

Unlike yeast, plants do not normally live in environments where external carbon sources are abundant, and one might therefore ask why they should have the ability to utilize them. One possibility is that this ability developed with multicellularity and the need to transport carbon from source to sink tissues. Alternatively, it is a much older trait that was present already in unicellular algae. In order to investigate how early in plant evolution the ability to absorb and metabolize external carbon sources emerged, we studied the growth of the charophyte microalga *K. nitens* on different carbon sources, both in the presence and in the absence of light. For these experiments, *K. nitens* was grown on plates supplemented with either 0.05 or 0.25 M of different carbon sources. The plates were incubated in constant light or in darkness for 1 month, after which growth was scored. As shown in Figure 1, we found that *K. nitens* is able to grow in the dark on all carbon sources tested except mannitol, which is a sugar that most organisms are unable to metabolize. However, *K. nitens* grew very poorly on 0.05 M galactose, and it failed to grow at the higher concentration of 0.25 M. The growth inhibitory effect of 0.25 M galactose was seen also in the presence of light, where growth does not require an external carbon source. This suggests that galactose is toxic to *K. nitens* at higher concentrations. We further note that higher concentrations of glucose and fructose also had a growth inhibitory effect (Figure 1). However, growth inhibition was not seen in the presence of 0.25 M sucrose, raffinose and mannitol, so it is not simply an osmotic effect. We conclude from these experiments that the ability to take up and metabolize external carbon sources is an ancient trait already present in simple charophyte algae.

### *Klebsormidium nitens* Has a Single Gene Encoding Hexokinase

**FIGURE 2:**
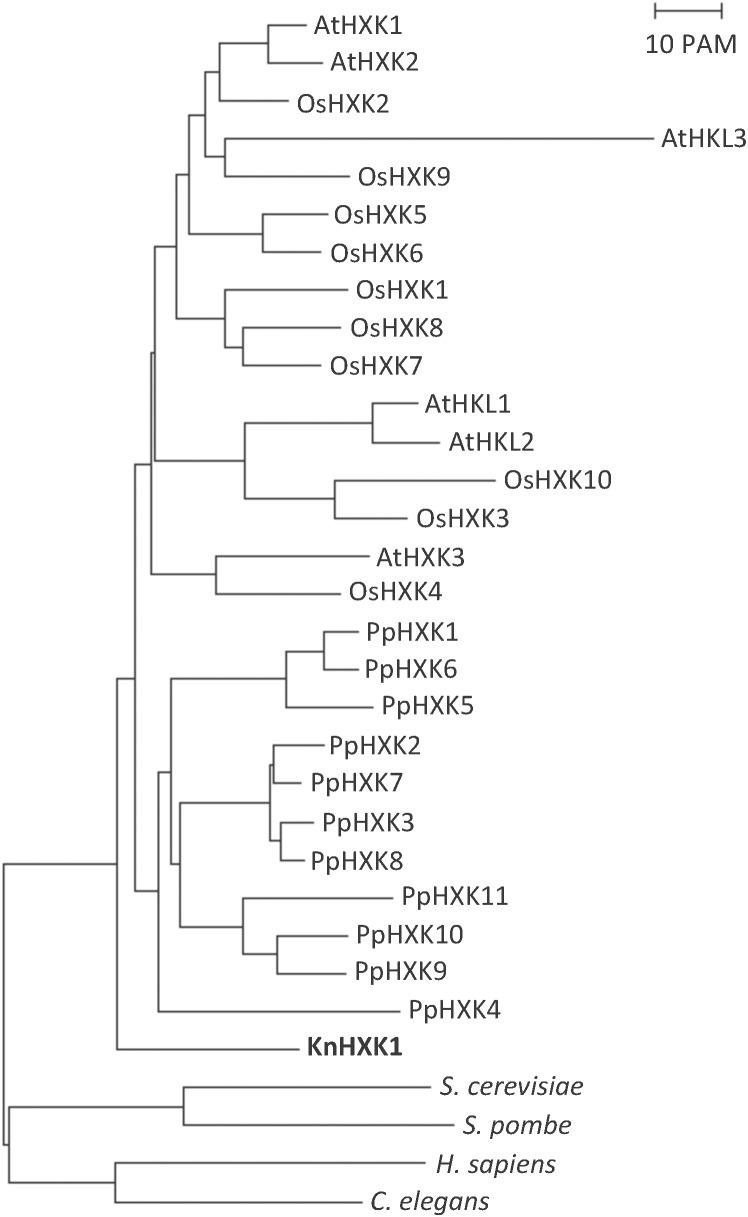
Phylogenetic tree of plant hexokinases. The tree is based on the alignment in Supplementary Figure S1, with the divergent N- and C-termini that failed to align in all hexokinases manually removed. The tree was calculated using the Neighbor-Joining method, as described in Materials and Methods. The bar represents a PAM value (percent accepted point mutations) of 10%. KnHXK1 is shown in bold.

A draft sequence of the *K. nitens* genome from the European strain SAG 52.91 (unpublished) revealed the presence of a single hexokinase encoding gene, which we named *KnHXK1*. The genome sequence of an American strain annotated as either *K. flaccidum* (Hori et al., 2014) or *K. nitens* (Ohtaka et al., 2017) also has a single hexokinase-encoding gene. The KnHXK1 protein has a predicted N-terminal sequence that is similar to the hydrophobic membrane anchor found in plant type B hexokinases, which localize to the mitochondrial membranes and the chloroplast envelope in *Physcomitrella* (Nilsson et al., 2011). The sequence of KnHXK1 was aligned to those of other plant hexokinases, as well as two fungal and two animal hexokinases (Supplementary Figure S1). The alignment was used to construct a phylogenetic tree rooted using fungal and animal sequences as an outgroup (Figure 2). We found that KnHXK1 is located at the expected position as the most basal branch among the plant sequences, consistent with an early split of land plants (embryophytes) from other streptophytes (charophytes) approximately 700 million years ago.

### KnHXK1 Localizes to Mitochondrial Membranes When Expressed in *Physcomitrella* Protoplasts

**FIGURE 3:**
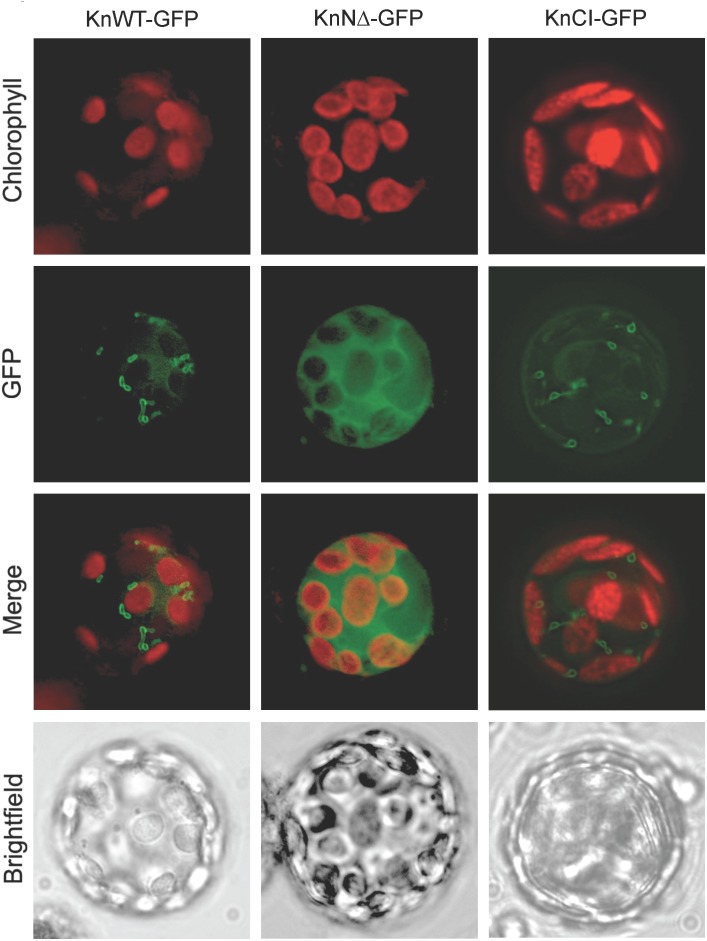
Intracellular localization of KnHXK1-GFP fusions. *Physcomitrella* protoplasts transiently expressing three different KnHXK1-GFP fusions are shown. Panels to the left show KnWT-GFP, which is intact KnHXK1 fused to GFP. Panels in the middle show KnNΔ-GFP, in which KnHXK1 lacks the N-terminal membrane anchor. The panels to the right show KnCI-GFP a catalytically inactive KnHXK1 containing two point mutations, one in the phosphoryl transfer site the other in the ATP binding site. GFP fluorescence is shown in green and chlorophyll auto-fluorescence in red.

To study the intracellular location of KnHXK1 we expressed three different KnHXK1-GFP fusions in *Physcomitrella* protoplasts: KnWT-GFP containing the full-length KnHXK1 coding sequence, KnNΔ-GFP producing a truncated protein without the predicted membrane anchor, and KnCI-GFP producing a protein with two point mutations rendering it catalytically inactive. GFP was added at the C-terminal end in order to minimize interference with correct folding of KnHXK1. The fusion constructs were expressed from the *CaMV35S* promoter. The constructs were transformed into *Physcomitrella* protoplasts after which the pattern of GFP fluorescence was studied. Chlorophyll autofluorescence was used as a chloroplast marker. For both KnWT-GFP and KnCI-GFP, the GFP signal localized to ring-like structures (Figure 3) that we have previously shown using MitoTracker staining to be mitochondria (Figure 5 in Nilsson et al., 2011). An identical localization has previously been observed in *Arabidopsis* for AtHXK1 and AtHXK2 (Giegé et al., 2003; Karve et al., 2008). When the membrane anchor was removed (KnNΔ-GFP) the GFP signal localized to the cytosol (Figure 3). We conclude that KnHXK1 is targeted to mitochondrial membranes, and that the N-terminal membrane anchor is needed for this localization. We further conclude that neither the catalytic site double mutant nor the N-terminal truncation destabilizes KnHXK1 or interferes with its expression in plant cells.

### Catalytically Active KnHXK1 Can Complement a Hexokinase-Deficient Yeast Strain for Growth on Glucose and Fructose

Several plant hexokinases have previously been shown to complement hexokinase-deficient yeast strains for growth on glucose or fructose when expressed from a yeast shuttle vector (Jang et al., 1997; Veramendi et al., 1999, 2002; Nilsson et al., 2011). To show that KnHXK1 is an enzymatically active hexokinases we tested if it could complement a *hxk1 hxk2 glk1* triple mutant yeast strain, which lacks all three yeast hexokinase genes (Hohmann et al., 1999). A cDNA encoding full-length KnHXK1 was cloned into pFL61, a yeast shuttle vector in which the insert is expressed from the *PGK* promoter (Minet et al., 1992). The resulting plasmid was named pKnWT. We also cloned an N-terminally truncated version of the KnHXK1 cDNA that lacked the membrane anchor into pFL61, generating pKnNΔ (Table 2). In addition, we made plasmids containing the KnHXK1 cDNA with and without membrane anchors as above, but containing two point mutations, one in the ATP binding site were the conserved glycine was mutated into an aspartic acid (G110D), and one in the phosphoryl transfer site were the conserved serine was mutated into an alanine (S184A). These are the same two point mutations that were reported to independently of each other abolish detectable catalytic activity but still support glucose signaling and glucose repression in *Arabidopsis* (Moore et al., 2003). We made a double mutant since experiments in yeast have shown that one of the two mutants (S158A in yeast Hxk2) is partially active with a relative *V*_max_ of 10% toward glucose and 50% toward fructose, compared to the wild type (Kraakman et al., 1999). We therefore reasoned that a double mutant might be more stringently inactive than either single mutant. The resulting constructs expressing the catalytically inactive double mutant of KnHXK1, with and without the membrane anchor, were named pKnCI and pKnNΔCI.

**FIGURE 4:**
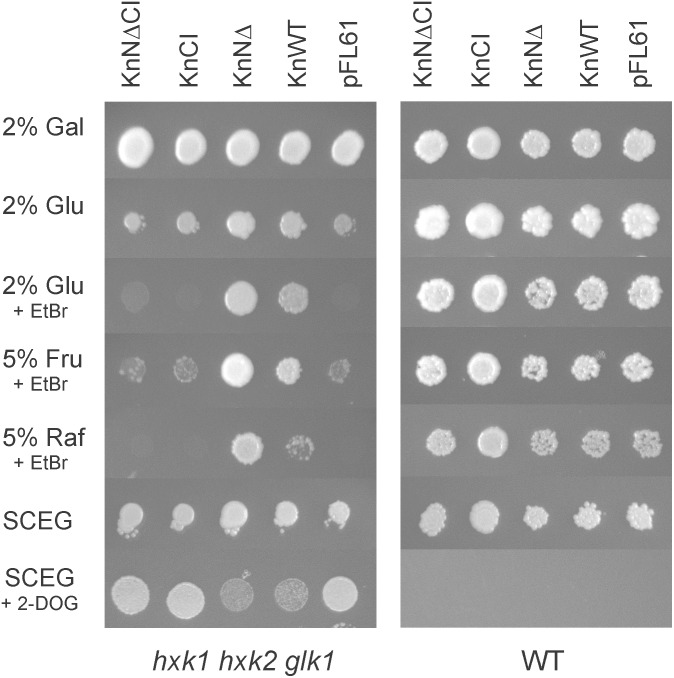
KnHXK1 can complement a *hxk1 hxk2 glk1* triple mutant yeast strain, lacking all hexokinase activity, both for growth on glucose or fructose and for glucose repression. The hexokinase-deficient strain is shown to the **left** and the wild type control strain to the **right**. The yeast strains were transformed with plasmids expressing the catalytically active KnHXK1 with membrane anchor (KnWT), without membrane anchor (KnNΔ) and catalytically inactive KnHXK1 with membrane anchor (KnCI) and without membrane anchor (KnNΔCI), as well as the empty vector (pFL61). The plates were uracil-less synthetic complete media containing 2% galactose (Gal), 2% glucose (Glu), 5% fructose (Fru), 5% raffinose (Raf), or 2% ethanol + 0.05% glucose (SCEG). Some plates also contained 1 μg/ml ethidium bromide (EtBr) in order to block oxidative growth, or 8 mg/ml 2-deoxyglucose (2-DOG) to induce glucose repression. Cells were spotted onto plates that were incubated for 4–5 days at 30°C prior to photography.

We found that full length KnHXK1 as well as KnHXK1 lacking the N-terminal membrane anchor could complement the hexokinase-deficient yeast strain for use of both glucose and fructose as a carbon source (Figure 4). This shows that KnHXK1 is an active hexokinase in yeast, and that KnHXK1 has a dual specificity for glucose and fructose. KnHXK1 also supports growth of the triple mutant strain *hxk1 hxk2 glk1* on raffinose. Both the wild type KnHXK1 and the mutant without membrane anchor support growth, but removing the membrane anchor seems to increase the hexokinase activity (Figure 4). In contrast, plasmids expressing the catalytic site double mutant KnHXK1, pKnCI, and pKnNΔCI, failed to complement the *hxk1 hxk2 glk1* strain for growth on glucose, fructose, or raffinose, as did the vector control (Figure 4). This confirms that the double mutation renders KnHXK1 enzymatically inactive.

### Catalytically Active KnHXK1 Can Restore Growth Inhibition by 2-Deoxyglucose in a Hexokinase-Deficient Yeast Strain

We went on to test if the different versions of KnHXK1 used in the yeast complementation study also could restore some aspects of glucose signaling in the hexokinase-deficient yeast strain. To this end, we grew the *hxk1 hxk2 glk1* yeast strain transformed with different plasmids on uracil-less ethanol/glycerol plates containing 2-deoxyglucose (Kraakman et al., 1999). Glucose inhibits growth on non-fermentable carbon sources, and the major yeast hexokinase, Hxk2, is needed for this response (Entian, 1980; Ronne, 1995; Johnston, 1999; Pelaez et al., 2010). 2-Deoxyglucose is a non-metabolized glucose analog that can trigger this response in the absence of glucose. Wild type yeast cells are therefore unable to grow on non-fermentable carbon sources in the presence of 2-deoxyglucose, but hexokinase-deficient yeast strains are able to do so.

The *hxk1 hxk2 glk1* strain, which unlike the wild type is able to grow on ethanol and glycerol in the presence of 2-deoxyglucose, was transformed with plasmids expressing wild type and mutant KnHXK1 proteins. Plasmids expressing the catalytically active versions of KnHXK1 partially restored growth inhibition on ethanol/glycerol in the presence of 2-deoxyglucose (Figure 4). In contrast, *hxk1 hxk2 glk1* cells expressing the catalytically inactive versions of KnHXK1 grew well on ethanol/glycerol media containing 0.8% 2-deoxyglucose, as did cells containing the empty vector pFL61 (Figure 4). We note that a higher concentration of 2-deoxyglucose (0.8%) was needed for hexokinase-dependent growth inhibition than in the study of Kraakman et al. (1999), where 0.02% was used. As expected, the wild type yeast strain expressing different KnHXK1 constructs was unable to grow on ethanol/glycerol in the presence of 2-deoxyglucose (Figure 4).

### KnHXK1 Cannot Restore Glucose Repression of Secreted Invertase in a *hkx2* Strain

**FIGURE 5:**
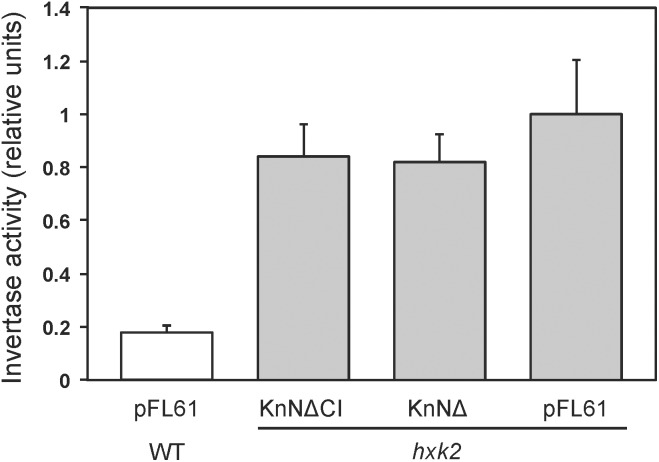
Glucose repression of secreted invertase in yeast. Secreted invertase activity was measured in either wild type (white bar) or *hxk2* mutant (gray bars) yeast cells grown in the presence of 2% glucose. The bars show averages of eight biological replicates. Error bars represent standard errors of means (SE). KnNΔ is the catalytically active KnHKX1 construct and KnNΔCI the catalytically inactive construct, both lacking the N-terminal membrane anchor in order to facilitate function in yeast.

One way in which hexokinase-dependent glucose signaling regulates the metabolism in yeast is by Mig1-dependent glucose repression, a process that requires both the major hexokinase Hxk2 and the glucose repressor Mig1. The established way of measuring glucose repression is by assaying secreted invertase, since the *SUC2* gene encoding invertase is regulated by glucose repression. We therefore transformed plasmids pKnNΔ and pKnNΔCI expressing catalytically active and inactive KnHXK1 into a *hxk2* knockout strain. The N-terminally truncated KnHXK1 was used since it seemed to work better in yeast (Figure 4). As controls, we also included wild type and *hxk2* strains transformed with the empty vector pFL61. As shown in Figure 5, we found that expression of secreted invertase was derepressed in the *hxk2* mutant, and neither the active nor the inactive KnHXK1 could restore glucose repression of secreted invertase. We conclude that KnHXK1 cannot complement the glucose repression deficient phenotype of the yeast *hxk2* mutant.

The ability of KnHXK1 to restore growth inhibition on non-fermentable carbon sources by 2-deoxyglucose (Figure 4), but its inability to restore glucose repression of secreted invertase (Figure 5), is somewhat surprising since sensitivity to 2-deoxyglucose frequently is used to assay glucose repression in yeast. However, Kraakman et al. (1999) found significant differences between the effects of glucose and 2-deoxyglucose on hexokinase-dependent repression of *SUC2*. Thus, the partially active HXK2-S158A mutant failed to repress *SUC2* in the presence of glucose, but still repressed it in the presence of 2-deoxyglucose (Figure 6 in Kraakman et al., 1999). From this result the authors concluded that the structural requirements for hexokinase-mediated signaling might vary in a substrate-dependent manner. This could also explain the differences between our results with 2-deoxyglucose and glucose.

### Catalytically Active KnHXK1 Can Restore Inhibition of Growth on High Glucose in an *Arabidopsis* Glucose-Insensitive Hexokinase Mutant

*Arabidopsis* HXK1 is involved in glucose signaling in addition to its role as a hexose phosphorylating enzyme (Moore et al., 2003). Similar to KnHXK1, AtHXK1 is a type B plant hexokinase that localizes to mitochondrial membranes. However, it also translocates to the nucleus in low amounts, where it is part of a complex that affects transcription (Cho et al., 2006). To investigate if KnHXK1 can replace the glucose signaling role of AtHXK1, we tested if KnHXK1 could complement the glucose insensitive *AtHXK1* mutant *gin2-1*. To this end, we made three different constructs that express wild type or mutant versions of KnHXK1 from the *CaMV35S* promoter. Constructs tested included catalytically active KnHXK1 (KnWT), a catalytically inactive KnHXK1 protein that had the same double mutation as used in previous yeast experiments (KnCI), and finally a catalytically active KnHXK1 in which the membrane anchor had been removed (KnNΔ). All constructs were transformed into the *Arabidopsis gin2-1* mutant. Three lines from each transformation were chosen for further studies.

**FIGURE 6:**
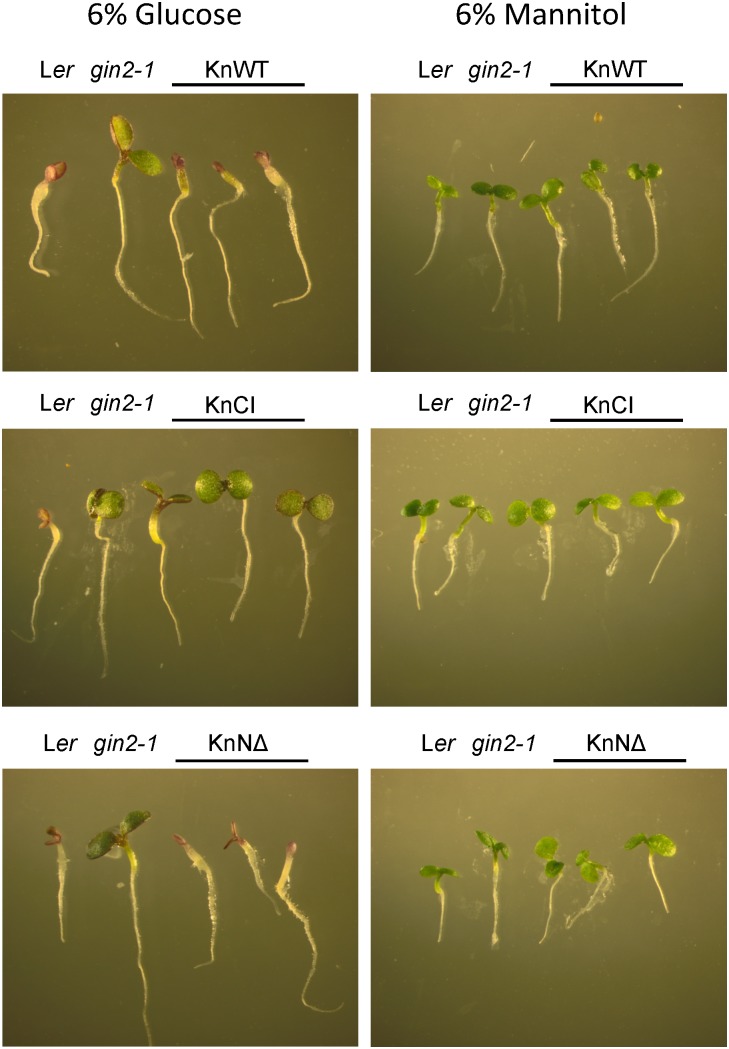
KnHXK1 can restore glucose inhibition of growth in the *Arabidopsis gin2-1* mutant. Transgenic *Arabidopsis gin2-1* seedlings are shown that were grown on 6% glucose **(left)** or 6% mannitol **(right)** for 6 days. Seedlings expressing wild type KnHXK1 (KnWT) are shown at the top, seedlings expressing the catalytically inactive KnHXK1 double mutant (KnCI) are shown in the middle, and seedlings expressing KnHXK1 in which the membrane anchor has been removed (KnNΔ) are shown at the bottom. The wild type (L*er*) and the untransformed *gin2-1* mutant are shown to the left in each panel.

In order to determine if wild type and mutant versions of KnHXK1 could restore glucose-sensing in the *gin2-1* mutant, we grew seedlings on plates containing either 6% glucose or 6% mannitol. We found that expression of wild type KnHXK1 restored a wild type response to glucose in 100% of the seedlings, including anthocyanin accumulation and short hypocotyl lengths (Figure 6 and Table 3). Lines expressing the membrane-anchor truncated KnHXK1 showed a wild type response to glucose in a majority of the seedlings, though the penetrance varied between different lines (Table 3). This suggests that the membrane anchor is not required for the glucose sensing mechanism in *Arabidopsis*. Lines expressing the catalytically inactive form of KnHXK1 did not behave like the wild type but instead retained the *gin2-1* mutant phenotype in 100% of the seedlings (Figure 6 and Table 3). It is unlikely that this reflects a problem in the synthesis, stability or folding of the catalytically inactive protein, since a GFP fusion to the mutant protein was expressed in *Physcomitrella* protoplasts and correctly targeted to the mitochondrial membranes (Figure 3). None of the phenotypes observed were due to osmotic stress since all transgenic lines as well as wild type and *gin2-1* seedlings were identical on plates containing 6% mannitol (Figure 6 and Table 3).

### Catalytically Active KnHXK1 Can Restore Normal Growth Under High Light in an *Arabidopsis* Glucose-Insensitive Hexokinase Mutant

**FIGURE 7:**
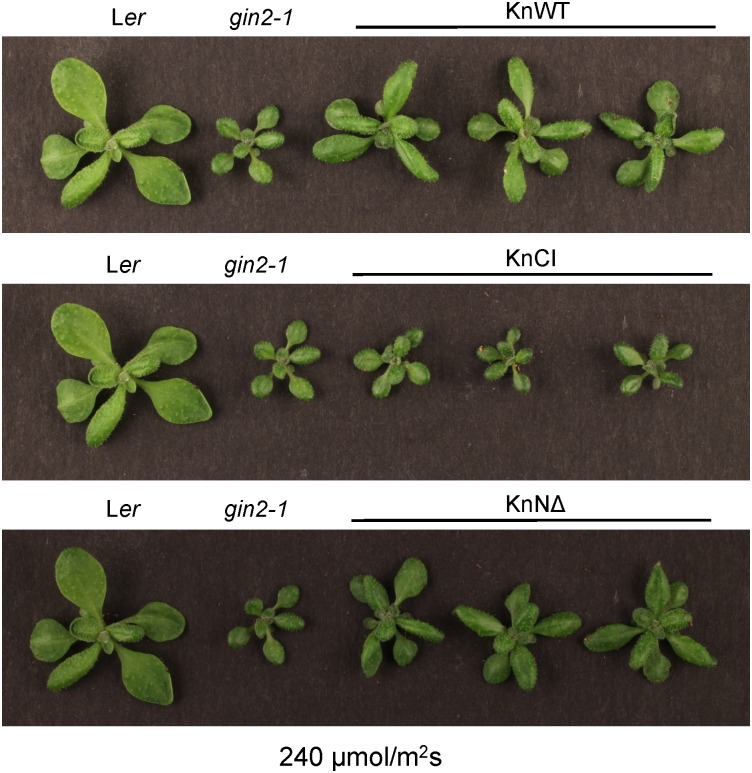
KnHXK1 can complement the *Arabidopsis gin2-1* mutant growth defect under high light conditions. Wild type (L*er*), *gin2-1* and transgenic *gin2-1* plants were grown for 18 days in high light (240 μmol/m^2^s) conditions. Three transgenic *gin2-1* plants expressing wild type KnHXK1 (KnWT) are shown at the *top*, three plants expressing the catalytically inactive KnHXK1 double mutant (KnCI) are shown in the *middle*, and three plants expressing KnHXK1 in which the membrane anchor has been removed (KnNΔ) are shown at the *bottom*. The wild type (L*er*) and the untransformed *gin2-1* mutant are shown to the left in each panel.

**FIGURE 8:**
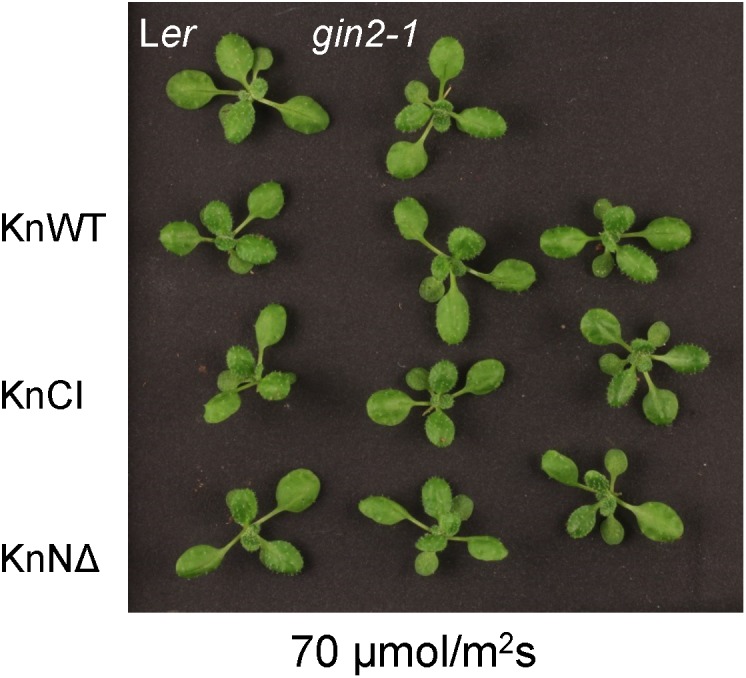
No effect of KnHXK1 or *gin2-1* under low light conditions. Wild type (L*er*), *gin2-1* and transgenic *gin2-1* plants were grown for 18 days in low light (70 μmol/m^2^s) conditions. The wild type (L*er*) and the untransformed *gin2-1* mutant are shown at the top. Three transgenic *gin2-1* plants expressing either wild type KnHXK1 (KnWT), the catalytically inactive KnHXK1 double mutant (KnCI), and KnHXK1 in which the membrane anchor has been removed (KnNΔ) are shown in the three rows below.

We proceeded to study the glucose signaling phenotypes using a second approach. The *gin2-1* mutant has a growth defect under high light conditions that leads to *gin2-1* plants becoming considerably smaller than wild type plants (Moore et al., 2003). We therefore tested if different versions of KnHXK1 could restore normal growth under high light conditions when expressed in the *gin2-1* mutant. Accordingly, we grew the transgenic lines expressing different versions of KnHXK1 under either high light (240 μmol m^-2^ s^-1^) or low light (70 μmol m^-2^ s^-1^) conditions. Under high light conditions the transgenic lines expressing the catalytically active versions of KnHXK1 restored the growth defect of *gin2-1*, though the plants were somewhat smaller than the wild type plants (Figure 7). However, similar to the experiment with glucose plates, lines expressing the catalytically inactive KnHXK1 double mutant did not restore the wild type phenotype but instead retained the growth defect of the *gin2-1* mutant (Figure 7). As expected, no differences were observed between the transgenic lines, the *gin2-1* mutant or the wild type under low light conditions (Figure 8).

### Catalytically Active KnHXK1 Can Restore Glucose Repression of Photosynthetic Genes in an *Arabidopsis* Glucose-Insensitive Hexokinase Mutant

**FIGURE 9:**
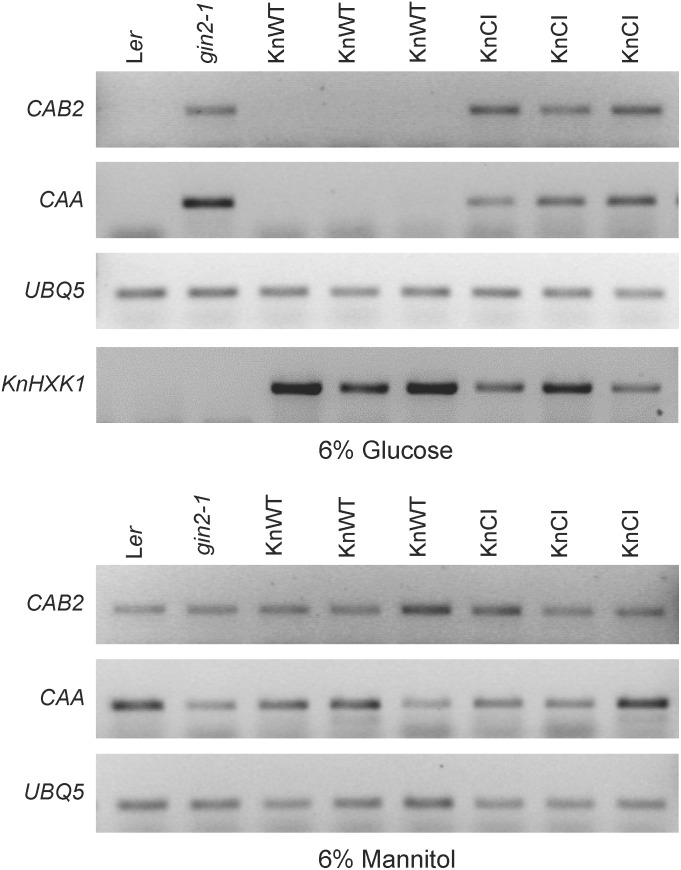
KnHXK1 can restore glucose repression of the *CAB2* and *CAA* genes in the *Arabidopsis gin2-1* mutant. The expression of the glucose repressed *CAB2* and *CAA* genes was monitored by RT-PCR in seedlings grown on glucose or mannitol. The constitutively expressed *UBQ5* gene was included as a control. Three lines expressing wild type KnHXK1 (KnWT) and three lines expressing the catalytically inactive double mutant KnHXK1 (KnCI) are shown. Also included were the wild type (L*er*) and the untransformed *gin2-1* mutant. The expression of the *KnHXK1* gene was also monitored on glucose in order to confirm that it was expressed in all transformed lines.

**FIGURE 10:**
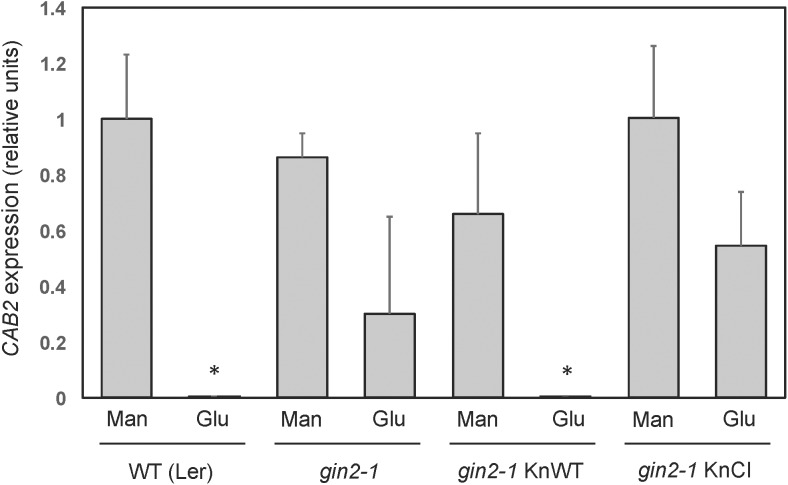
Quantification of *CAB2* expression in *Arabidopsis* KnHXK1 transformants by qPCR. The mRNA levels of the glucose repressed *CAB2* gene were quantified by qPCR in seedlings grown on glucose or mannitol. Expression of the constitutively expressed *UBQ4* gene was used as an internal control. Three biological replicates each of the wild type (*Ler*), the *gin2-1* mutant, *gin2-1* transformed with the wild type *KnHXK1* construct (KnWT) and *gin2-1* transformed with the catalytically inactive double mutant *KnHXK1* (KnCI) were analyzed. The bars show the average level of *CAB2* expression relative to the wild type (*Ler*) on mannitol in three biological replicates, after normalization of each sample against the *UBQ4* expression level. The error bars show standard deviations. The *asterisks* indicate values that differ significantly (*p* < 0.01) from the wild type on mannitol using the two-tailed *t*-test. The *gin2-1* KnWT values on glucose and mannitol also differ significantly from each other (*p* < 0.05).

Similar to yeast, a number of *Arabidopsis* genes are transcriptionally repressed by glucose, and this response is also deficient in the *gin2-1* mutant (Rolland et al., 2002; Moore et al., 2003). To test if KnHXK1 is able to restore the transcriptional response to glucose when expressed in the *gin2-1* mutant, we used RT-PCR to examine mRNA levels of the two glucose repressed photosynthetic genes *CAA* and *CAB2*. We found that the wild type version of KnHXK1 expressed in the *gin2-1* mutant restored glucose repression of these genes, thus behaving similar to the wild type (Figure 9). In contrast, the catalytically inactive KnHXK1 mutant did not restore glucose repression, and these transformants thus behaved similar to the *gin2-1* mutant (Figure 9). As a control we also monitored expression of the two genes on 6% mannitol. We found that neither the wild type nor the *gin2-1* mutant, nor any of the transgenic lines showed repression of the genes under these conditions. To verify that the wild type and double mutant KnHXK1 constructs were properly expressed, we also analyzed their expression by RT-PCR in the lines grown on glucose (Figure 9). These results were further confirmed by quantitative RT-PCR (qPCR) using the *CAB2* gene. As shown in Figure 10, we found that expression of *CAB2* is completely repressed on glucose in the wild type (*Ler*) and in the *gin2-1* line transformed with the wild type *KnHXK1* gene (KnWT), but not in the *gin2-1* line transformed with the catalytically inactive *KnHXK1* gene (KnCI). We conclude that the defect in glucose repression in the *gin2-1* mutant can be complemented by KnHXK1, but only in its catalytically active form.

## Discussion

We have cloned and characterized the *KnHXK1* gene of the charophyte alga *K. nitens*. *KnHXK1* encodes a type B plant hexokinase (Olsson et al., 2003; Nilsson et al., 2011), and similar to other type B hexokinases it has an N-terminal mitochondrial targeting peptide, which targeted a KnHXK1-GFP fusion protein to mitochondrial membranes when transiently expressed in *Physcomitrella* protoplasts (Figure 3). There are several types of plant hexokinases: the type A hexokinases that possess chloroplast transit peptides that localize them to the chloroplast stroma, the type B and D hexokinases that have N-terminal membrane anchors and localize to the mitochondrial membranes and chloroplast envelopes, and finally the type C hexokinases that lack both membrane anchor and transit peptide and remain in the cytosol (Olsson et al., 2003; Nilsson et al., 2011). The subcellular localizations of different hexokinases probably reflect different functions. The type B hexokinases have been proposed to be important in the supply of pyruvate to mitochondria. In *Arabidopsis*, almost all enzymes in the glycolytic pathway are associated with the cytosolic face of the mitochondrial membrane (Giegé et al., 2003). A likely reason why KnHXK1 localizes to mitochondrial membranes is therefore that it functions in supplying pyruvate to the mitochondria.

Since KnHXK1 is the only hexokinase that we found to be encoded by the *K. nitens* genome, it seems plausible that the type B hexokinase is the ancestral enzyme from which other types of plant hexokinases evolved. This would suggest that the primary function of plant hexokinases originally was to provide pyruvate to the mitochondria. However, it should be noted that in the moss *Physcomitrella*, the type A hexokinase PpHXK1 which localizes to the chloroplast stroma is responsible for 80% of the glucose phosphorylating activity (Olsson et al., 2003). This raises the question why *K. nitens* does not have a type A hexokinase. It is possible that the type A hexokinases arose as an adaptation in early land plants, since all land plants that have been examined possess type A hexokinases. This in turn raises the question what the advantage of having a hexokinase in the chloroplast stroma is, and why this should be more important to land plants. We have previously suggested that the type A hexokinases function in mobilization of glucose from starch stored in the chloroplasts (Olsson et al., 2003; Nilsson et al., 2011). This process may be more important in multicellular plants where carbon is redistributed between sink and source tissues. Alternatively, the switch from day to night requires a faster mobilization of glucose in land plants, which are more exposed to diurnal temperature changes.

Our findings that KnHXK1 can restore both glucose signaling (Figures 6, 7) and glucose repression (Figures 9, 10) in the *Arabidopsis gin2-1* mutant and that the catalytic activity of KnHXK1 is necessary for this restoration differ from previous results on AtHXK1 (Moore et al., 2003) and its orthologs in rice (Cho et al., 2009), but are consistent with the finding that the ability of the cytoplasmic rice hexokinase OsHXK7 to complement *gin2-1* depends on its catalytic activity (Kim et al., 2016). A trivial explanation for these differences would be if some catalytic activity is needed for all glucose signaling, and if the AtHXK1 single mutants (and their rice orthologs) are leaky, with some residual catalytic activity. We chose to make a double mutant in KnHXK1 in order to minimize the risk for leakiness, since studies in yeast have shown that the first mutant (S158A in yeast Hxk2) is partially active with a relative *V*_max_ of 10% for glucose and 50% for fructose (Kraakman et al., 1999), and since an HA-tagged version of the second mutant, AtHXK1-G104D, appeared to have a residual activity of about 5% of the HA-tagged wild type protein (Figure S3 in Moore et al., 2003). However, both mutant proteins were completely inactive when purified as His-tagged proteins (Feng et al., 2015). Furthermore, the results of Kim et al. (2016) with OsHXK7 cannot be explained by leakiness since both single mutants in that case did abolish glucose signaling. We therefore think that leakiness of single mutants is an unlikely explanation for the differences between our results and those of previous studies (Moore et al., 2003; Cho et al., 2009).

The question then arises why the ability of plant hexokinases to mediate glucose sensing and glucose repression is dependent on their catalytic activity in some cases (KnHXK1 and OsHXK7) and independent in other cases (AtHXK1, OsHXK5, and OsHXK6). First, we note that this difference does not seem to reflect these two groups of hexokinases being involved in different physiological responses. It has been suggested that different hexokinase-dependent responses to glucose exist in plants, some of which are dependent on the catalytic activity and some of which are not (Xiao et al., 2000). Thus, the glucose-induced *Arabidopsis* defense genes *PR1* and *PR5* are induced in plants that overexpress either AtHXK1 or yeast ScHXK2, which suggests a dependency on hexokinase activity rather than on plant-specific glucose signaling (Xiao et al., 2000). In contrast, a different set of genes is repressed by the AtHXK1-dependent glucose signaling pathway, e.g., photosynthetic genes such as *CAA*, and these genes are not repressed by expression of ScHXK2 (Xiao et al., 2000). However, we note that *CAA* is repressed by both OsHXK7 (Kim et al., 2016) and KnHXK1 (Figure 9) when expressed in the *gin2-1* mutant. Moreover, other phenotypes of the *gin2-1* mutant such as glucose insensitivity of plant seedlings and growth inhibition under high light conditions are also complemented by both OsHXK7 (Kim et al., 2016) and KnHXK1 (Figures 6, 7). We conclude that KnHXK1 and OsHXK7 mediate the same physiological response to glucose as AtHXK1, OsHXK5, and OsHXK6, even though the former two but not the latter three are dependent on their catalytic activity.

Interestingly, a cyanobacterial glucokinase, cGlk, from *Synechocystis* could also complement the *gin2-1* defects in both glucose-responsive growth phenotypes and glucose repression of the *CAB1* and *RBCS* genes (Ryu et al., 2008). This is puzzling given the reported inability of yeast ScHXK2 to substitute for AtHXK1 (Xiao et al., 2000) since the prokaryotic cGlk is much more distantly related to plant hexokinases than is ScHXK2 (Kawai et al., 2005). One would therefore expect ScHXK2 to be able to provide any function in plants that cGlk can provide. However, we note that published results with ScHXK2 in plants involved expression of ScHXK2 in either wild type plants (Xiao et al., 2000) or maize protoplasts (Yanagisawa et al., 2003). The ability of ScHXK2 to complement *gin2-1* does not seem to have been tested. It also remains to be shown if the ability of cGlk to complement *gin2-1* depends on its catalytic activity.

It therefore seems likely that increased glucose phosphorylation and a resulting increase in metabolic intermediates or ATP could be the common mechanism responsible for the ability of KnHXK1, OsHXK7, and cGlk, and perhaps other hexokinases, to complement the glucose signaling and glucose repression defects of *gin2-1*. The fact that catalytically inactive mutants of AtHXK1 can trigger the same response (Moore et al., 2003) shows that a glucose signaling pathway that does not require hexokinase catalytic activity has evolved in at least some plants, but apparently the two pathways function in parallel and the same response can still be elicited by increased glucose phosphorylation. The question then arises why the other two catalytically active hexokinases in *Arabidopsis*, AtHXK2, and AtHXK3, cannot substitute for AtHXK1 in glucose signaling. This could be due to localization (AtHXK3 is a type A hexokinase that localizes to the plastid stroma) and/or expression levels. We note that KnHXK1, OsHXK7, and cGlk were all expressed from the constitutive CaMV 35S promoter. Possibly, a high and stable level of expression is needed to sustain glucose signaling through increased glucose phosphorylation.

**FIGURE 11:**
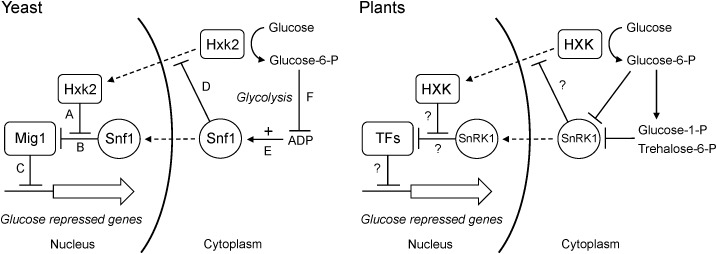
Dual roles of hexokinase in glucose repression. The situation in yeast is shown to the **left**. **(A)** The hexokinase Hxk2 forms a trimeric complex with the Mig1 repressor and the Snf1 protein kinase in the nucleus. **(B)** In the absence of Hxk2, Snf1 phosphorylates Mig1, which is then excluded from the nucleus. However, Hxk2 masks the Snf1 target site in Mig1, thus protecting it from phosphorylation. **(C)** In the presence of Hxk2, Mig1 therefore stays in the nucleus and represses the glucose repressed genes. **(D)** Nuclear localization of Hxk2 is regulated by Snf1, which phosphorylates Hxk2 causing it to be excluded from the nucleus. **(E)** Snf1 is activated by ADP, which signals a low energy level. **(F)** Hexokinase phosphorylates glucose and activates glycolysis, which reduces the ADP level and thus inactivates Snf1. This in turn activates Mig1-dependent glucose repression even in the absence of a nuclear Hxk2. The situation in plants is shown to the **right**. Less is known about nuclear interactions, but some hexokinases and SnRK1s are present in the nucleus and have been shown to associate with both transcription factors and target promoters. It is therefore possible that they could participate in regulatory interactions similar to those in yeast. As in yeast, SnRK1 is inactivated by increased glucose phosphorylation, though the mechanism involves inhibition of SnRK1 by sugar phosphates rather than its activation by ADP. It is not known if SnRK1 regulates nuclear transport of plant hexokinases. Nuclear transport is indicated by *dashed arrows*, positive effects by *solid arrows* and negative effects by *solid T-bars*.

What mechanism could link glucose phosphorylation to changes in gene expression? An obvious candidate, as suggested by Halford et al. (1999a), is the Snf1 kinase, known as AMPK in animals and SnRK1 in plants. Snf1 is a global energy sensor that is activated by low energy conditions and replenishes the energy level by inducing catabolic energy-generating pathways and repressing anabolic pathways. Animal AMPK is activated by AMP and yeast Snf1 by ADP (Mayer et al., 2011). SnRK1 is not activated by either AMP or ADP, but is instead inhibited by glucose-6-P, glucose-1-P and in particular trehalose-6-P (Zhang et al., 2009; Martinez-Barajas et al., 2011; Nunes et al., 2013; Emanuelle et al., 2016). However, the end result is the same: the kinase is activated during low energy conditions, and increased glucose phosphorylation by hexokinase will inactivate it. SnRK1 could therefore play a similar role in plants as Snf1 does in yeast, where hexokinase-dependent inhibition of Snf1 through increased glucose phosphorylation and a resulting decrease in ADP contributes to glucose repression even in the absence of a nuclear hexokinase (Figure 11). Such a model would be consistent with the findings that hexokinase and SnRK1 mutants have opposite phenotypes both in the moss *Physcomitrella* (Olsson et al., 2003; Thelander et al., 2004, 2007) and in *Arabidopsis* (Baena-Gonzalez et al., 2007).

There are several ways to test this model. First, if glucose repression mediated by KnHXK1, OsHXK7 and cGlk is dependent on SnRK1, a knockout mutant devoid of all SnRK1 activity should block this effect. This could be tested in *Physcomitrella*, where a *snf1a snf1b* double knockout is viable (Thelander et al., 2004). Second, if nuclear SnRK1 functions downstream of hexokinase similar to Snf1 in yeast (Figure 11), a SnRK1-less mutant should be deficient in expression of glucose repressed genes such as *CAA* and *CAB*. We note that photosynthetic genes was one group of genes that was induced by overexpression of KIN10, the *Arabidopsis* SnRK1 (Figure S1 in Baena-Gonzalez et al., 2007), but the reverse question, i.e., to what extent the expression of these and other hexokinase-regulated plant genes is dependent on SnRK1 remains to be answered. Third, the effect of expressing ScHXK2 in plants should be re-examined. In particular, the question if ScHXK2 can complement the *gin2-1* phenotypes needs to be addressed. As noted above, published results on ScHXK2 in plants deal only with its expression in wild type plants and protoplasts. Fourth, the model predicts that the cyanobacterial glucokinase cGlk also should depend on its catalytic activity for the ability to complement *gin2-1*, similar to KnHXK1 and OsHXK7.

Finally, we note that catalytically active hexokinases might inhibit Snf1 and/or SnRK1 by other mechanisms that do not involve sugar phosphates or the glycolytic flux. This could explain our finding that KnHXK1 expressed in a hexokinase-deficient yeast strain can restore growth inhibition on non-fermentable carbon sources by 2-deoxyglucose (Figure 4), which is not metabolized beyond the phosphorylation step and thus does not increase the glycolytic flux. However, results obtained with glucose analogs should be interpreted with caution since the structural requirements for hexokinase-mediated signaling may vary in a substrate-dependent manner (Kraakman et al., 1999).

## Conclusion

We found that the charophyte hexokinase KnHXK1 can complement the glucose signaling and glucose repression phenotypes of a glucose-insensitive *Arabidopsis* hexokinase mutant. We further found that these effects depend on the catalytic activity of KnHXK1, unlike the case for AtHXK1 and its rice orthologs, but similar to rice OsHXK7. We interpret these findings as evidence of dual roles for plant hexokinases in glucose signaling: (1) by specific interactions with transcription factors that work only between angiosperms (e.g., *Arabidopsis* and rice), and (2) by effects on hexose phosphorylation and/or the glycolytic flux, which can be provided also by a distantly related hexokinases such as KnHXK1.

## Author Contributions

MU, DME, and HR conceived the study, designed the experiments, and wrote the manuscript. MU, G-ZH, and DME did the experimental work. All authors read and approved the manuscript.

## Conflict of Interest Statement

The authors declare that the research was conducted in the absence of any commercial or financial relationships that could be construed as a potential conflict of interest.

## Abbreviations

GFP: green fluorescent protein
PCR: polymerase chain reaction
RT-PCR: reverse transcriptase-polymerase chain reaction
