# Supplementary material for: The Ability of a Charophyte Alga Hexokinase to Restore Glucose Signaling and Glucose Repression of Gene Expression in a Glucose-Insensitive Arabidopsis Hexokinase Mutant Depends on Its Catalytic Activity
Source: Front Plant Sci. 2018 Dec 20;9:1887. doi: 10.3389/fpls.2018.01887 (PMC6306471; doi:10.3389/fpls.2018.01887)
Supplement: Supplementary file 1 [file Image_1.pdf]

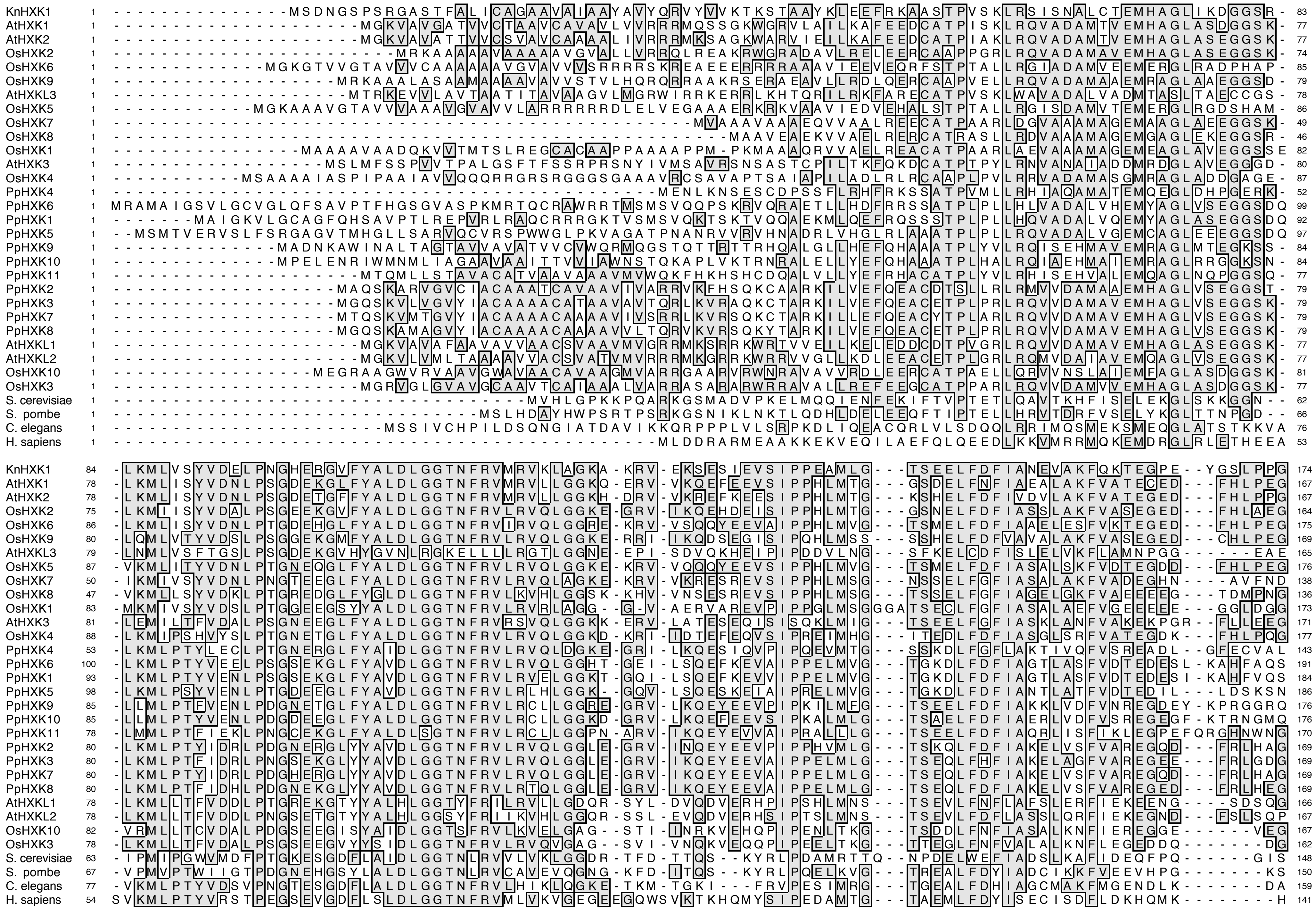
KnHXK1
AtHXK1
AtHXK2
OsHXK2
OsHXK6
OsHXK9
AtHXKL3
OsHXK5
OsHXK7
OsHXK8
OsHXK1
AtHXK3
OsHXK4
PpHXK4
PpHXK6
PpHXK1
PpHXK5
PpHXK9
PpHXK10
PpHXK11
PpHXK2
PpHXK3
PpHXK7
PpHXK8
AtHXKL1
AtHXKL2
OsHXK10
OsHXK3
S. cerevisiae
S. pombe
C. elegans
H. sapiens

KnHXK1	175	QK-RELGFTFSFPCNQT---AIDGGSLVRWTKGFKVSGTVGKDVVKLLTEALTRK-G-VDMRV-AALVNDTVGTLAGGRY-NNNDVMVGLILGTGSNAC	265
AtHXK1	168	RQ-RELGFTFSFPVKQT---SLSSGSLIKWTKGFSIEEAVGQDVVGALNKALERV-G-LDMRI-AALVNDTVGTLAGGRY-YNPDVVAAVILGTGTNAA	258
AtHXK2	168	RQ-RELGFTFSFPVKQL---SLSSGTLINWTKGFSIDDTVDKDVVGELVKAMERV-G-LDMLV-AALVNDTIGTLAGGRY-TNPDVVVAVILGTGTNAA	258
OsHXK2	165	RQ-RELGFTFSFPVKQT---SIASGTLINWTKGFSIDETVGEDVVTELTKALERQ-G-LDMKV-TALINDTIGTLAGGRY-DDNDVIAAVILGTGTNAA	255
OsHXK6	176	RQ-RELGFTFSFPVHQT---SISSGTLIKWTKGFSINGTVGEDVVAELSRAMERQ-G-LDMKV-TALVNDTVGTLAGGRY-VDNDVAAAVILGTGTNAA	266
OsHXK9	170	TQ-RELGFTFSFPVKQK---SLASGTLIKWTKSFAIDEMVGKDVVAELNMAIRSQ-G-LDMKV-TALVNDTVGTLAAGRY-VNHDTIAAVILGTGSNAA	260
AtHXKL3	166	EV-KNLGFTLTRSVEQIGSHSISSI-HRKSLANDD-DEKVLKDLVNDMNESLETH-G-LKIRMNTALVDNTIGELAGGRY-YHKDTVAAVSLGMGTNAA	258
OsHXK5	177	RQ-RELGFTFSFPVSQT---SISSGTLIKWTKGFSINDAVGEDVVSELGKAMERQ-G-LDMKI-AALVNDTVGTLAGGRY-ADNSVVAAIILGTGTNAA	267
OsHXK7	139	RQ-RELGFTFSFPVRQT---SIASGTLIKWTKAFSIDDAVGEDVVAELQMAMEKQ-G-LDMRV-SALTNDTVGTLAAGSY-YDEDIVVGVILGTGSNAA	229
OsHXK8	137	KK-KELGFTFSFPVRQR---SVASGTLVKWTKAFSIDDAVGEDVVAELQTAMVKQ-G-LDMHV-AALINDAVGTLAGARY-YDEDVVAGVIFGTGTNAA	227
OsHXK1	174	E--RELGFTFSFPVHQT---SIASGTLIRWTKAFAVDDAIGEDVVAALQAAMSER-G-LDMRV-SALINDTVGTLAAGSY-YDEDVVAAVILGTGTNAA	263
AtHXK3	172	RK-RELGFTFSFPVKQT---SIDSGTLSKWTKGFKVSGMEGKNVVACLNEAMEAH-G-LDMRV-SALVNDGVGTLAGARY-WDEDVMVGVILGTGTNAC	262
OsHXK4	178	RK-RELGFTFSFPVNQT---SIDSGILIKWTKGFAVSGTAGKDVVACLNAAMERQ-G-LDMRV-SALVNDTVGTLAGARY-WDDDVMVAVILGTGTNAC	268
PpHXK4	144	NQKRDIGFTFSFPVNQT---KVNGGSINAWTKGFSISDGVGEDVVDQLEIALADM-GSVNTKV-VCLVNDTVGTLAQCRY-WNDDAMVGVILGTGSNAC	236
PpHXK6	192	GKVRESGFAFSFPVRQT---SVKSGIVIHWTKGFKVDDAVGKDIVKQFQDAISRS-G-HQIAI-SALVNDTVGTLAGGRFNFEEETMIGCIIGTGTNAC	284
PpHXK1	185	GKTRESGFAFSFPVRQT---SVKSGIVIHWTKGFKVDDAVGKDIVKQFQDAISRS-N-HQIMI-SALVNDTVGTLAGGRFNFDEETMIGCIIGTGTNAC	277
PpHXK5	187	KH-REAGFAFSFPVRQT---SVKSGNVIQWTKGFKIDDAIGKDIVKQFQDAISRS-G-HDVEI-SALVNDTVGTLAGGRYNFQEETMIGCILGTGTNAC	278
PpHXK9	177	QTVRELGLTFSFPVKQT---SVRSGVLIQWSKGFLVADGVGADVVALLQRAINRQHG-PKIEV-VVLVNDTVGTLAGGRY-WNEDAMVGMILGTGTNAC	269
PpHXK10	177	QTVREMGLTFSFPVKQQ---SVKSGAIIQWSKGFDIADGVGADVVALLQSAINRQRG-PKIEV-AVLVNDTVGTLAGGRY-WNEDVMVGMILGTGTNAC	269
PpHXK11	171	HQIRELGLTISFPICQT---SHNTGILIKWTEGFKIADGVGKDVVAMLQSAMDRQKG-FQIRV-AVLINDTVGTMAGGHY-WNDDVMVGVILGTNTNAC	263
PpHXK2	170	QQ-REIGFTFSFPVDQT---AVNSGKLLQWTKGFKVNDAIGQDVVAALQRSIESL-G-HKMRI-SALINDTVGTLAGGRY-WNNDVMIGVILGTGTNAC	260
PpHXK3	170	QS-REIGFTFSFPCKQT---AVNSGTLLQWTKGFKVNDAIGQDVVAALQKCIERL-G-CKMRI-AALVNDTVGTLAGGRY-WNNDVMIAVILGTGTNAC	260
PpHXK7	170	QN-REIGFTFSFPVKQT---AVNSGTLLQWTKGFKVNDAVGEDVVAALQRGIERR-G-YKMRI-AALINDTVGTLAGGRY-WNNDVMIGVILGTGTNAC	260
PpHXK8	170	QS-REIGFTFSFPCKQT---AVNSGTLLQWTKGFKVNDAIGQDVVAALQGSIERR-G-YKMRI-AALVNDTVGTLAGGRY-WNNDVMIAVILGTGTNAC	260
AtHXKL1	167	VR-RELAFTFSFPVKHT---SISSGVLIKWTKGFEISEMVGQDIAECLQGALNRR-G-LDMHV-AALVNDTVGALSLGYY-HDPDTVAAVVFGTGSNAC	257
AtHXKL2	168	LK-RELAFTFSFPVKQT---SISSGVLIKWTKGFAISEMAGEDIAECLQGALNKR-G-LDIRV-AALVNDTVGALSFGHF-HDPDTIAAVVFGTGSNAC	258
OsHXK10	168	---RALGFTFSFPVRQT---SISSGTLIRWTKEFSIEEAVGKDVAQCLNEALARN-G-LNMKV-NVLVNNTVGTLALGHY-YDDDTVAAVIIGAGTNAC	256
OsHXK3	163	K--MALGFTFSFPVRQI---SVSSGSLIRWTKGFSIRDTVGRDVAQCLNEALANC-G-LNVRV-TALVNDTVGTLALGHY-YDEDTVAAVIIGSGTNAC	252
S. cerevisiae	149	EP-IPLGFTFSFPASQN---KINEGILQRWTKGFDIPNIENHDVVPMLQKQITKR-N-IPIEV-VALINDTTGTLVASYY-TDPETKMGVIFGTGVNGA	239
S. pombe	151	QN-LEIGFTFSYPCVQR---SINDASLVAWTKGFDIDGVEGESVGPLLSAALKRV-GCNNVRL-NAILSDTTGTLVASNY-ASPGTEIGVIFGTGCNAC	242
C. elegans	160	QK-LPLGFTFSFPCEQE---GLTKGKLVTWTKGFKASGVEGVDVVTLLHEACHRR-KDIDIDV-VALLNDTVGTLMACAF-QENSCQIGVIVGTGTNAC	251
H. sapiens	142	KK-LPLGFTFSFPVRHE---DIDKGILLNWTKGFKASGAEGNNVVGLLRDAIKRR-GDFEMDV-VAMVNDTVATMISCYY-EDHQCEVGMIVGTGCNAC	233

KnHXK1	266	YVEDVHDIPKWTGG-PVESGE----MVINMEWGDFYSAH---LPITDVDRELDEVSVNPGEQPFEKLISGMYLGEIVRRVLLQLAEEGEIFEG-VTEKL	355
AtHXK1	259	YVERATAIPKWHGL-LPKSGE----MVINMEWGNFRSSH---LPLTEFDHTLDFESLNPGEQILEKIISGMYLGEILRRVLLKMAEDAAFFGDTVPSKL	349
AtHXK2	259	YVERAHAIPKWHGL-LPKSGE----MVINMEWGNFRSSH---LPLTEYDHSLDVDSLNPGEQILEKIISGMYLGEILRRVLLKMAEEAAFFGDIVPPKL	349
OsHXK2	256	YVERANAIPKWHDL-LPKSGD----MVINMEWGNFRSSH---LPLTEFDQALDAESLNPGEQVYEKLISGMYLGEIVRRVLLKMAEEASLFGDEVPPKL	346
OsHXK6	267	YVEHANAIPKWTGL-LPRSGN----MVINMEWGNFKSER---LPRSDYDNALDFESLNPGEQIYEKMISGMYLGEIVRRILLKLAHDASLFGDVVPTKL	357
OsHXK9	261	YIDHADAIPKWHGS-LPKSGN----MVINMEWGNFKSSH---LPLTEFDQELDAESLNPGKQVYEKSISGMYMGELVRRILLKMAQETRIFGDNIPPKL	351
AtHXKL3	259	YIEQAQEISRWKSA-IREPQE----IVVSTEWGDFRSCH---LPITEFDASLDAESLNPGHRIFEKMVSGRYLGEIVRRVLLKMSEESALFGDTLPPKL	349
OsHXK5	268	YVENANAIPKWTGL-LPRSGN----MVINTEWGSFKSDK---LPLSEFDKAMDFESLNPGEQIYEKLISGMYLGEIVRRILLKLAHDAALFGDVVPSKL	358
OsHXK7	230	YLEKANAIPKLEGE-LPKSGN----MVINTEWGNFSSSC---LPITEYDEALDKESLNPGEQIFEKLISGMYLGEIVRRVLLKISLQSSIFGNLDQTKL	320
OsHXK8	228	YVEKANAIPKWEGE-LPNSGD----MVINMEWGNFYSSH---LPVTEYDEALDKESLNPGEQIYEKLTSGMYLGEIVRRVLLKLSLQSGIFGSIDNSKL	318
OsHXK1	264	YVEDATAIAKLHPSQLPASNT----MVINTEWGSFASPC---LPLTEFDEALDQESLNPGEQTYEKLISGMYLGEIVRRVLLKISSRCPSLLGGAGE-L	354
AtHXK3	263	YVEQKHAIPKLR-S-KSSSGT----TIINTEWGGF-SKI---LPQTIFDLEMDETSLNPGEHLYEKMISGMYLGEIVRRVLLHMCETSDLFGHFAPAKL	351
OsHXK4	269	YIQRTEAIPKLQHL-KLETGN----TIINTEWGAF-SDG---LPLTEFDREMDDESINPGEQIFEKTISGMYLGEIVRRVLVKMAEVSDLFGHSFPKKL	358
PpHXK4	237	YVERAAAISCWSSP-PEADDL----TVVNIEWGNFCSEL---LPRTFADEGLDADSLNPGQQEFEKMIGGMYLGEILRRVLLKMAEDTGLFGSEIPERL	327
PpHXK6	285	YVERADSVQKWADP-LPKSGQ----MVINMEWGNFHSPF---LPRTFADDIVDKDSVNPGDQWFEKMISGMYLGEIVRLVLARMAEEAQLFGGSPPAKL	375
PpHXK1	278	YVERADAVHKWDEP-LPKSGE----MVINMEWGNFRSPY---LPRTFADETVDKDSVNPGDQWFEKMISGMYLGEIVRLVLARMAKEAELFGGNVPVKL	368
PpHXK5	279	YVERADAVKKWKEA-LPKSGE----MVINLEWGNFRSPW---LPRTFADDEVDKESVNPGDQWFEKMVSGMYLGEIVRHMLLRLAEEATLFGDTVPEKL	369
PpHXK9	270	YVER-D-LPAHA---NSSTGE----MVINMEWAGFWSSH---LPRTYADEQLDSESVNPGQAGFEKMIGGMYLGEIVRRVLFKMAGEAALFGDEIPHKL	356
PpHXK10	270	YVEH-D-LPSHV---QSGSGE----MIINMEWGGFWSSH---LPRTFADEQLDKESLNPGQAGYEKMIGGMYLGEIVRRVLLRMEKEASLFGGPVPSKL	356
PpHXK11	264	YVEC-N-LPEDI---QTKSGK----MVIYMEWGRFWSSH---LPRTYIDEQLDNESVNPGDRGFEKMTGAMYLGEIVRRVLARMAQEANLFGDSVPTKL	350
PpHXK2	261	YVERAEAVSKWGGE-IPKSGQ----MVINMEWGNFWSSH---LPRTYVDESLDNESLNPGEYGFEKMISGMYLGDCVRRVLVRMAQQAGIFGPRVPHRL	351
PpHXK3	261	YVERAESISKWTGE-LPKSGQ----MVINMEWGNFWSSH---LPRTYVDELLDSESLHPGEYGFEKMISGMYLGDCVRRVLVRMAQEAGIFGPHVPHTL	351
PpHXK7	261	YVERAEAVSKWAGD-IPKSGE----MVINMEWGNFWSSH---LPRTYVDESLDNESLNPGEYGFEKMISGMYLGDCVRRVLVRMAQEACIFGTPVPHKL	351
PpHXK8	261	YVERAESISKWGGE-LPKSGQ----MVINMEWGNFWSSH---LPRTYVDELLDNESLNPGEYGFEKMISGMYLGDCVRRVLVRMAQQAGIFGPHVPHKL	351
AtHXKL1	258	YLERTDAIIKCQGL-LTTSGS----MVVNMEWGNFWSSH---LPRTSYDIDLDAESSNANDMGFEKMISGMYLGDIVRRVILRMSEDSDIFGPISPV-L	347
AtHXKL2	259	YLERTDAIIKCQNP-RTTSGS----MVVNMEWGNFWSSR---LPRTSYDLELDAESMNSNDMGFEKMIGGMYLGDIVRRVILRMSQESDIFGPISSI-L	348
OsHXK10	257	YIERNDAIIKSLGR-VTNSER----TVVNVEWGSFRPPQ---IELTPYDICFNNETWNYYDQGFEKMISGVYLGEIARLVFQKMAEESDIFGTAVDG-L	346
OsHXK3	253	YIERTDAIIKCQGL-LTNSGG----MVVNMEWGNFWSSH---LPRTPYDILLDDETHNRNDQGFEKMISGMYLGEIARLVFHRMAQESDVFGDAADS-L	342
S. cerevisiae	240	YYDVCSDIEKLQGK-LSDDIPPSAPMATNCEYGSFDNEHVV-LPRTKYDITIDEESPRPGQQTFEKMSSGYYLGEILRLALMDMYKQGFIFKNQDLSKF	336
S. pombe	243	YIEKFSEIPKLHKY-DFPEDMN---MIINCEWCDFDNQHVV-LPRTKYDVAIDEESPRPGLQTYEKMIAGCYLGDILRRILLDLYEQGALFNGQDVTKI	336
C. elegans	252	YMERLDRIPKLAGY-VDEHGVTPEEMIINTEWGAFGDDGALDFLRTEWDEVVDRESINPGQHLYEKMISGMYMGECARVVLEDLAKQGLLFGGNSDA-I	348
H. sapiens	234	YME---EMQNVELV-EGDEGR----MCVNTEWGAFGDSGELDEFLLEYDRLVDESSANPGQQLYEKLIGGKYMGELVRLVLLRLVDENLLFHGEASEQL	324

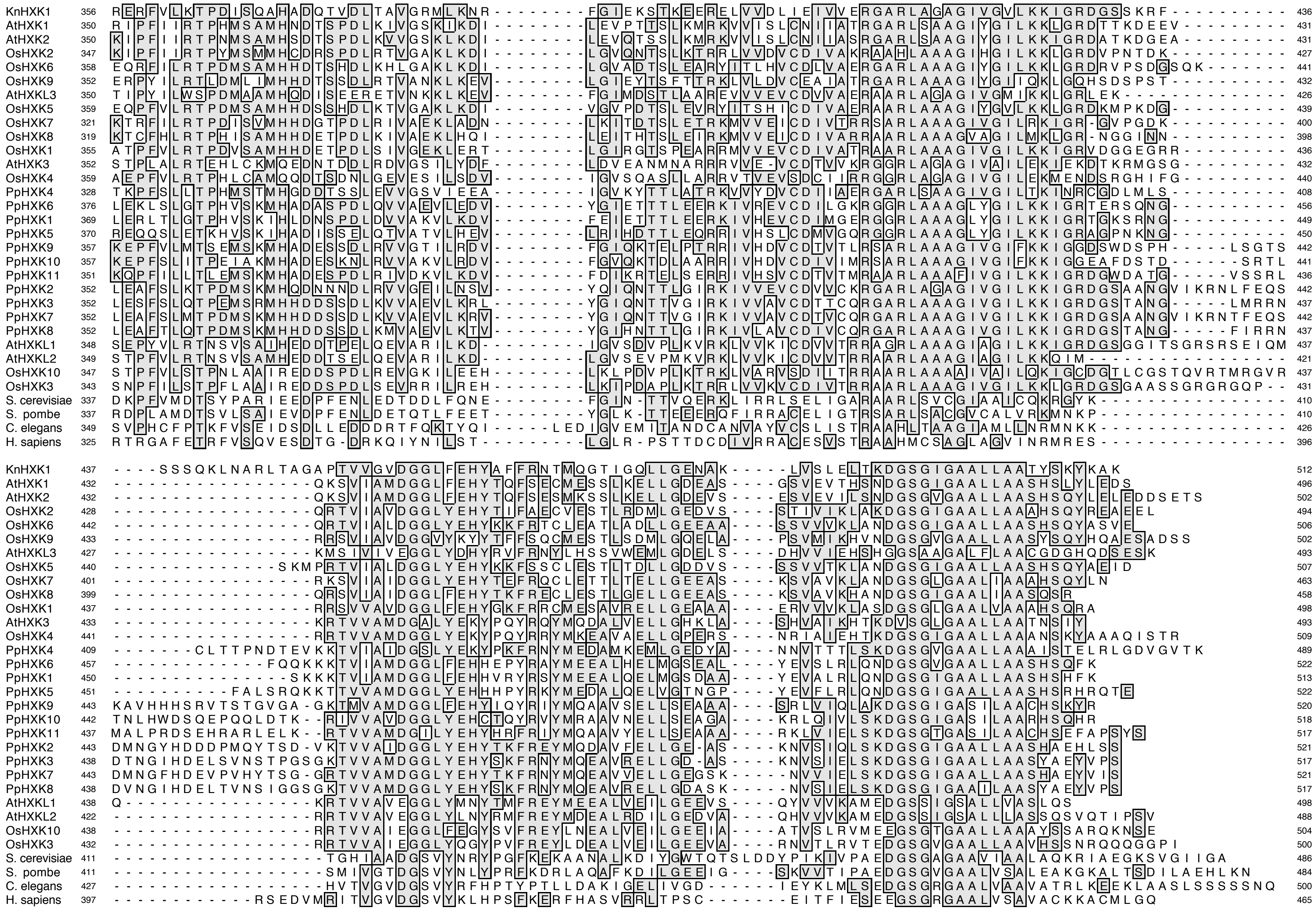

FIGURE S1. Multiple alignment of KnHXK1 to other hexokinases. The sequence of KnHXK1 was aligned to all available hexokinases in *Arabidopsis*, rice and *Physcomitrella*, and to one hexokinase each from two animals (*H. sapiens* and *C. elegans*) and two fungi (*S. cerevisiae* and *S. pombe*) as outgroups. Conserved amino acid residues are enclosed in boxes.
